# Lattice Engineering Novel 2D Monolayer in Zinc Pnictides

**DOI:** 10.1021/acsomega.5c05775

**Published:** 2025-10-24

**Authors:** Dinesh Thapa, Seong-Gon Kim

**Affiliations:** † Department of Mathematics and Physics, 2722 Thomas More University, Crestview Hills, Kentucky 41017, United States; ‡ Department of Physics and Astronomy, 5547 Mississippi State University, Starkville, Mississippi 39762, United States

## Abstract

In this work, the structural, electronic, and thermodynamic stabilities in the novel two-dimensional monolayer (2D-ML) structure of IIB–VA zinc pnictides, ZnX (X = As, Sb, Bi), have been systematically investigated via lattice engineering. We utilize the geometries of 3D bulk structures of ZnX in orthorhombic symmetry with space group *Pbca*(*No*.61) as parental material to model three different 2D monolayers of ZnX, denoted as 2D-(L1, L2, and L3). Their total relative energies and stabilities have been investigated and compared with the 2D monolayer geometries of tetragonal, hexagonal (planar honeycomb), and wurtzite (puckered honeycomb) symmetries. The spin-polarized density functional theory (DFT) with plane wave basis sets has been employed throughout the calculations, with the hybrid HSE06 functional to get an accurate description of thermodynamic stability and electronic band gap values consistent with experimental data. Our calculations suggest that the 2D-L1 monolayer with rectangular symmetry obtained from the lattice relaxation of quasi-layered rhomboid rings (Zn_2_X_2_) dramatically represents the ground-state monolayer in zinc pnictide compounds. While the 2D-ML in tetragonal geometry is energetically competitive in ZnSb or favorable in ZnBi, it shows slight dynamical instability, reinforcing that 2D-L1 is the only structure found to be dynamically stable at zero strain. The feasibility of the most stable 2D-L1 monolayer has been realized with its dynamical stability, as manifested by the absence of imaginary frequencies in phonon dispersion curves, together with mechanical and thermal stabilities via *ab initio* molecular dynamics (AIMD). The band gap becomes wider in the 2D-L1 monolayer compared to its bulk counterparts. The nature of the band gap is slightly indirect in 2D-L1 monolayer of ZnAs, whereas it is direct in that of ZnSb, and ZnBi. Notably, the indication of a negative Poisson’s ratio in the most stable 2D-L1 monolayer in ZnAs signifies its auxetic property. Our theoretical findings provide a potential synthesis route for novel 2D-ML structures in ZnX, which have yet to be experimentally synthesized.

## Graphical abstract

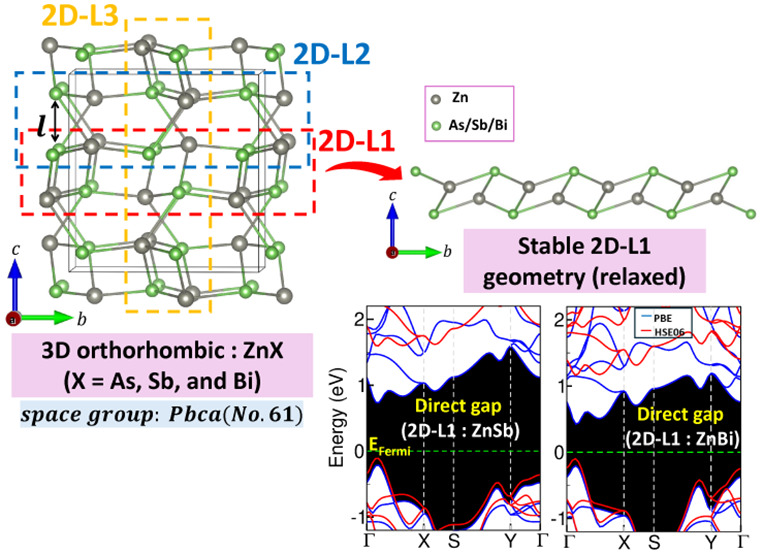

## Introduction

The evolution of functional two-dimensional (2D) materials began with the isolation of single-layer graphene in 2004, with its appealing electronic feature of Dirac cones, where electrons behave as massless or relativistic particles.^1,2^ The 2D materials (single or few atomic layers) have attracted the attention of many theoretical and experimental researchers due to their unique and easily tunable electrochemical, optical, and mechanical properties, with tremendous applications in nanoengineering, optoelectronics, and spintronics.^3–7^ Multilayer-thick 2D materials such as transition metal dichalcogenides (TMDCs), nanoclay, and MXenes form a potential platform for heterostructure engineering. The 2D materials that belong to TMDCs such as MX_2_ (M = Mo, W; X = S, Se, and Te), and metal oxides such as ZnO, are considered intriguing materials due to their exceptional properties, including a direct band gap in the visible range, high exciton binding energy, and large carrier mobility.^8–10^ In addition, the electrochemical, magnetic, and optical properties of such 2D materials can be enhanced to be applied in broader areas of material science by incorporating point defects,^11–14^ functionalizing with organic molecules^15–17^ and by providing excess charge on the surfaces, substantially lowering their work function. Recently, charged 2D metal surfaces with extremely low work functions have been extensively utilized in electrocatalytic reduction. The 2D copper (Cu) metal surface with excess surface charge exhibits high activation energy compared to the neutral metal surface that inhibits oxidative corrosion of the metal.^18^ Similarly, the 2D van der Waals (vdW) electride crystals, [RECl]^2+^·2e^–^ has become a benchmark for providing experimental opportunities to explore the fundamentals of magneto-electric and quantum properties.^19^ Furthermore, 2D nanoclay structures doped with Fe–Mg impurities are found to play a significant role in tuning the ferromagnetic (FM), ferrimagnetic (FEM), and antiferromagnetic (AFM) order for biomedical implications, when interfaced with unnatural amino acids.^16^

Earlier research confirmed that 3D bulk ZnSb with orthorhombic symmetry is a promising material due to its thermoelectric and strong anisotropic transport properties.^20–22^ The peculiarity behind the favorable thermoelectric performance of ZnSb is its low lattice thermal conductivity.^23^ Because of the increasing demand for ZnSb as a thermoelectric material, it seems important to obtain more theoretical insights into the structural, electronic, and phase stabilities of its congeners ZnAs and ZnBi in bulk, as well as in their 2D monolayers, carving simple pathways for their experimental synthesis. Moreover, its relative phase stability, high charge carrier mobility, and Seebeck coefficient have recently grabbed the attention of several researchers.^24,25^ However, ZnAs is metastable at ambient pressure and congruently melts into its stable phases ZnAs_2_ and Zn_3_As_2_. So, the experimental synthesis of equiatomic ZnAs can be realized only through the pressure-induced decomposition of ZnAs_2_ or Zn_3_As_2_ or from the direct reaction of the elements under high-pressure and -temperature conditions. Both ZnAs and ZnSb compounds in orthorhombic symmetry are electron-poor semiconductors (EPSs) with only seven valence electrons per formula unit, that feature simultaneous multicenter and two-center bonding.^23^ Further, the electrical transport properties of ZnAs are different from those of ZnSb, which is mainly attributed to the larger band gap of ZnAs (∼0.9 eV) compared to that of ZnSb (∼0.5 eV). The experimental Seebeck coefficient of ZnAs, ∼27 μV/K, is rather small compared to that of ZnSb, ∼300 μV/K, at room temperature. This often indicates that ZnAs is a poor thermoelectric material. However, both compounds indicate very similar vibrational properties owing to their temperature dependence of heat capacity, and the interaction of optical and acoustic phonons explains the low thermal conductivity of both ZnAs and ZnSb.^26^

This research has been carried out to explore a new two-dimensional monolayer (2D-ML) beyond existing ones with exceptional structural, electronic, and mechanical properties. Various 3D bulk materials especially van der Waal structures exhibit promising possibilities to give rise to a prominent 2D material via mechanical exfoliation.^27–29^ This type of transformation of the crystal structure from 3D to 2D is a key factor for new device architectures, switching the properties of existing materials via dimensional reduction. This paper deals with the theoretical investigation of a novel 2D monolayer with structural stability, together with its intriguing electronic properties in IIB–VA zinc pnictides, ZnX (X = As, Sb, and Bi). To account for this fact, the structural, electronic, and thermodynamic stabilities of the 2D-ML structures derived from orthorhombic 3D bulk ZnX, with space group *Pbca*(*No*.61) have been analyzed in this study, and their stabilities have been compared with other possible tetragonal and hexagonal geometries.

Some attempts have been made in the past to exfoliate the 2D monolayer of ZnSb both theoretically and experimentally; however, the mechanically and thermodynamically stable 2D structures of these ZnX pnictides have not yet been declared. Specifically, in a 2D-ZnSb study, Song et al. synthesized sp^2^ hybridized layered 2D-ZnSb via the dimensional manipulation of sp^3^ 3D-ZnSb (bulk) and the selective etching of alkali metals. The authors reported that the free-standing stable 2D monolayer of ZnSb was hardly achieved in its wurtzite (puckered honeycomb) as well as hexagonal (planar honeycomb) structures with sp^2^ hybridized geometry.^30^ Another theoretical study performed by Bafekry et al. carried out DFT simulation on the anisotropic puckered honeycomb structure of 2D ZnSb with *P*3*m*1 space group, which was found to be unstable in its pristine form; however, it showed stability via surface functionalization by semifluorination and chlorination.^31^ Since the reported structures of pristine 2D-ZnSb monolayers lack structural stability, it necessitates the search for stable geometries of 2D ZnSb and its congeneric binary compounds, ZnAs and ZnBi. Herein, we studied six possible 2D monolayer structures of ZnX (X= As, Sb, and Bi), in which three of them are derived from the 3D bulk structure of ZnX (space group *Pbca*), denoted as 2D-(L1, L2, and L3) monolayers, and the other three are in the tetragonal structure of FeS type with space group *P*4/*nmm*(*No*.129), the planar honeycomb structure of hexagonal boron nitride (h-BN) with space group *P*6̅*m*2­(No.187), and the puckered honeycomb structure of boron phosphide (BP) type with space group *P*3*m*1­(*No*.156). Our theoretical calculations elucidate the fact that the ground-state geometry of the 2D monolayer in ZnX exists in rectangular symmetry in the 2D-L1 monolayer, as manifested by its mechanical, thermal, and dynamical stabilities compared to other 2D configurations. To the best of our knowledge, no optimized structures and electronic properties for the 2D monolayer with its stable 2D-L1 geometry in ZnX have yet been reported. Further, our novel 2D structures in ZnX could serve as a good starting point for training machine learning (ML) models, enhancing validation and feasibility of our lowest-energy (equilibrium) 2D structures.

## Computational Methods

The first-principle quantum mechanical calculations are performed using the spin polarized density functional theory (DFT) based on the projector augmented wave (PAW) method^32^ within the generalized gradient approximation (GGA)^33^ implemented in Vienna Ab initio Simulation Package (VASP).^34,35^ The similar methods using VASP have been employed to perform DFT calculations in previous works to get the accurate description of the structural and electronic properties of various topological materials.^36–38^ The geometrical structures of ZnX (X = As, Sb, and Bi) in 3D orthorhombic symmetry (space group *Pbca*) are retrieved from the *Materials Project* database.^39^ The pseudopotential valence electron configurations used during the calculations for Zn, As, Sb, and Bi are 3d^10^4s^2^, 4s^2^4p^3^, 5s^2^5p^3^, and 6s^2^6p^3^, respectively. The calculations utilize the Perdew–Burke–Ernzerhof (PBE) exchange-correlation functional^40^ within the inclusion of spin–orbit coupling (SOC) and without (w/o) SOC effect in the most stable 3D and 2D models; PBE with van der Waals (vdW) corrections in the 3D bulk; and the hybrid HSE06 functional^41^ to improve the electronic band gap in 3D and the most stable 2D structures. The plane-wave energy cutoff of 525 eV is used for all the calculations. The 3D bulk and 2D monolayers of ZnX are fully relaxed including the lattice vectors and the atom positions during the structural optimization using the conjugated gradient method until the total energy is converged numerically to less than 1.0 × 10^–6^ eV per unit cell and the force on each atom is less than 10^–3^ eV/Å. We perform the systematic lattice engineering of the 2D monolayers, relaxing all the atom positions and lattice vectors to find the ground state structures in monolayers in six different crystallographic symmetries including the three 2D-(L1, L2, and L3) derived from 3D bulk ZnX, tetragonal, hexagonal (planar honeycomb), and wurtzite (puckered honeycomb) structures. The Brillouin zone (BZ) integration for the geometrical relaxations is accurately performed using Monkhorst-pack grid with a k-mesh of 8 × 6 × 6 for the 3D bulk ZnX (X = As, Sb, and Bi). The BZ sampling for all the 2D monolayers is so chosen that it satisfies the inverse relation between the real space and the Fourier transform of the real space lattice vectors. The vacuum length ∼30.0 Å is set in all the 2D-ML structures to safely avoid interlayer interaction. The standard DFT method significantly underestimates the Kohn–Sham (KS) band gaps compared with that obtained in the experiment. Therefore, the hybrid Hartree–Fock (HF) method, which describes the exchange energy using a mixture of the nonlocal HF exchange $\left(E_{x}^{\mathrm{HF}}\right)$ and GGA exchange functional $\left(E_{x}^{\mathrm{GGA}}\right)$ ^42^ is used to accurately predict the electronic band gap^43^ and crystal formation energies using the PBE optimized geometries. Since, the hybrid functionals are computationally expensive, the short-range functionals such as the screened hybrid functional HSE06 are considered computationally effective and the standard hybrid functional. The exchange correlation energy in the standard HSE06 can be calculated as^44^

1 $$E_{\mathrm{XC}}^{\mathrm{HSE}06}=\frac{1}{4}E_{X}^{\mathrm{HF},\mathrm{SR}}\left(\omega \right)+\frac{3}{4}E_{X}^{\mathrm{PBE},\mathrm{SR}}\left(\omega \right)+E_{X}^{\mathrm{PBE},\mathrm{LR}}\left(\omega \right)+E_{C}^{\mathrm{PBE}}$$

where the screening parameter ω = 0.2 Å^–1^ corresponds to the HSE06 method. Here, ω defines the range separation of the electron–electron interaction into a short-range (SR) and long-range (LR) part, and $E_{C}^{\mathrm{PBE}}$ is the PBE correlation energy, which is expressed in eq 2.

2 $$E_{C}^{\mathrm{PBE}}=\int d^{3}r\rho \left(\mathrm{r⃗}\right)\epsilon_{\mathrm{XC}}^{\mathrm{PBE}}(r_{s}(\mathrm{r⃗},s\left(\mathrm{r⃗},\zeta \left(\mathrm{r⃗}\right)\right)))$$

where $\epsilon_{\mathrm{XC}}^{\mathrm{PBE}}$ is the exchange correlation energy equivalent to the homogeneous electron gas in the spin polarized case related to the spin orientation. $\zeta =\frac{\rho_{↑}-\rho_{↓}}{\rho }$ , is the spin polarized electron density where ρ^↑^(*r⃗*), ρ^↓^(*r⃗*) are the spin-up and spin-down electron density, respectively. $r_{s}={\left(\frac{4}{3}\pi \rho \right)}^{-\frac{1}{3}}$ is the Weigner Seitz radius. Here, $s=\frac{\left|\nabla \rho \right|}{2k_{F}\rho }$ is the reduced density gradient and $k_{F}={\left(3\pi^{2}\rho \right)}^{1/3}$ .^40^ Additionally, van der Waals (vdW) correction optB86b-vdW of Klimeš et al. in the 3D bulk systems is introduced, to get the nonlocal interactions between the atoms.^19,45^ However, the vdW correction has not been implemented in the 2D systems, owing to their large vacuum thickness, where the interlayer vdW interaction is negligible. Further, the spin–orbit coupling (SOC) effect has been implemented to get the relativistic corrections in 3D bulk ZnSb and the most stable 2D structure, using the noncollinear scheme in VASP. The SOC term, ĤSOC=γL⃗^S⃗^ is added to the Kohn–Sham Hamiltonian, where γ is the spin–orbit coupling strength, L⃗^ is the orbital angular momentum operator, and S⃗^ is the electron spin operator (Pauli matrices).^46^

Phonon frequency calculations are performed within the framework of the supercell approach using density functional perturbation theory (DFPT) as implemented in the phonopy code,^47^ in which the force constants are calculated using VASP. The supercell is so chosen to ensure that there is no self-interaction of the displaced atom with itself due to the periodic boundary condition (PBC). Within the DFPT approach, the total energy is numerically converged to less than 10^–5^ eV per supercell and the force on each atom is less than 10^–2^ eV/atom. To achieve accuracy in phonon calculations, a 2 × 2 × 2 supercell consisting of 128 atoms is adopted for the 3D bulk structures of ZnX with orthorhombic symmetry. Similarly, in 2D-ML structures, the phonon dispersion curves (PHDCs) are calculated using a 4 × 4 × 1 supercell consisting of 128 atoms in 2D-L1, 2D-L2, and 2D-L3 monolayers, a 4 × 4 × 1 supercell consisting of 64 atoms in the 2D tetragonal structure of ZnX (X = As, Sb, and Bi), and a 6 × 6 × 1 supercell consisting of 72 atoms for the 2D monolayer of hexagonal and wurtzite structures of ZnSb. All geometrical structures and charge density difference (Δρ) plots are generated using the VESTA program.^48^ The mechanical properties of the 3D and 2D ZnX structures have been studied using the energy-strain approach implemented in the VASPKIT code.^49^

## Results and Discussions

In this study, the structural, electronic, and thermodynamic stabilities of the two-dimensional monolayer (2D-ML) in ZnX (X= As, Sb, Bi), together with the most stable 3D bulk structure in its stable orthorhombic symmetry with space group *Pbca*(*No*.61) have been explored using spin-polarized density functional theory (DFT). This section is divided into several categories: (I) ground-state observables in 3D bulk ZnX, (II) crystal symmetry of 2D-ML ZnX derived from 3D bulk, (III) structural stability in 2D-ML ZnX, (IV) dynamical stability in 3D bulk and 2D-ML structures, (V) calculation of exfoliation energy, (VI) ground-state observables in 2D-L1 monolayer, and (VII) electronic transitions in 2D-ML structures.

### Ground-State Observables in 3D Bulk ZnX

#### Geometrical Structure

The stoichiometric 3D bulk geometry of ZnX (X = As, Sb, and Bi) with orthorhombic symmetry (*a* ≠ *b* ≠ *c*, and α = β = γ = 90°) is isostructural with the CdSb type, containing 8 formula units in the unit cell, where (Zn, X) atoms occupy the Wyckoff positions (8c, 8c) in the crystal lattice.^50–52^ The structure consists of edge-sharing ZnX_4_ tetrahedra that in turn are connected via common corners. Each atom in the ZnX structure attains a peculiar 5-fold coordination by one like and four unlike neighbors. At the same time, each atom is also part of planar rhomboid rings (parallelograms) constituting a Zn_2_X_2_ structure, in which two layers of rhomboid rings are related by a gliding operation and form the quasi-layered structure along the *c* and *b* directions. These rhomboid rings contain a shorter Zn–Zn diagonal compared to X–X, which is a consequence of edge-sharing ZnX_4_ tetrahedra. Further, the chains of rhomboid rings are interconnected in the structure through the two shortest Zn–X bonds of 2.49 Å in ZnAs, 2.69 Å in ZnSb, and 2.79 Å in ZnBi. There are six distinct nearest-neighbor distances in ZnX connected within the rhomboid ring. The rhomboid ring represents a four-center, four-electron (4c4e) bonded entity that is connected via 2c2e bonds to neighboring rings. 2c2e are the bonds, which are not involved in forming the rhomboid rings.^23^ Here, the experimentally synthesized stable phase of 3D bulk ZnBi has not been realized yet; however, we used the similar orthorhombic phase of ZnBi in this study to compare its lattice parameters and electronic band structures with its congeneric ZnAs and ZnSb phases. The relaxed geometrical structure of the bulk ZnX is shown in Figure 1, and a rhomboid ring is shown encircled with a black dotted line in Figure 1c. ZnX has seven valence electrons per formula unit: 2 from Zn and 5 from X atoms. Such electron-poor valence often indicates a semiconducting nature in ZnAs and ZnSb. Further, the semiconducting nature in ZnAs and ZnSb is attributed to sp^3^ hybrid orbitals in the tetrahedron, classifying them as electron-poor semiconductors.^53^ The relaxed geometrical structures of 3D bulk ZnX reveal the fact that the PBE-optimized lattice parameters in ZnAs and ZnSb agree well with experimental and other calculated results. Herein, we present our calculated lattice parameters for 3D bulk ZnBi in orthorhombic symmetry, which has not yet been reported. The van der Waals interaction energy (Δ*E*_vdW_) per atom in ZnX has been calculated using the relation *E*_vdW_ = *E*_w*/*o–vdW_ *– E*_vdW_, where *E*_w/o–vdW_ is the total energy per atom for the bulk optimized with the PBE functional without (w/o) vdW correction, and *E*_vdW_ is the total energy per atom for the bulk geometry optimized with vdW correction. The calculated Δ*E*_vdW_ values for ZnAs, ZnSb, and ZnBi are 1.983, 2.548, and 3.770 meV/atom, respectively. This infers that the 3D ZnX is a non-vdW structure, where the interlayer atoms interact through weak van der Waals force. The lattice parameters (*a*, *b*, and *c*), equilibrium volume (*V*_o_), and the separation (*l*) along the *c*-axis between the quasi-layered rhomboid rings in the 3D bulk ZnX (X = As, Sb, and Bi) using PBE and PBE + vdW are reported in Table 1.

**Table 1:** The Optimized Lattice Parameters (*a*, *b*, and *c*), Equilibrium Volume (*V*_o_), Interlayer Separation (*l*) along the *c*-Axis in the 3D Bulk ZnX Using PBE and PBE + vdW, Together with Experimental and Other Calculated Results

ZnX	Properties	PBE	PBE + vdW	Expt.^26,54^	Other calculated^23^
ZnAs	*a* (Å)	5.750	5.727	5.679, 5.673	5.751
	*b* (Å)	7.336	7.284	7.277, 7.275	7.342
	*c* (Å)	7.653	7.580	7.559, 7.557	7.659
	*V*_o_ (Å^3^)	322.818	316.203	312.384, 311.885	323.392
	*l* (Å)	1.781	1.764	-	-
ZnSb	*a* (Å)	6.284	6.252	6.218	6.287
	*b* (Å)	7.817	7.755	7.741	7.824
	*c* (Å)	8.226	8.131	8.114	8.229
	*V*_o_ (Å^3^)	404.078	394.225	390.555	404.780
	*l* (Å)	2.020	1.998	-	-
ZnBi	*a* (Å)	6.536	6.487	-	-
	*b* (Å)	8.113	8.030	-	-
	*c* (Å)	8.501	8.375	-	-
	*V*_o_ (Å^3^)	450.779	436.259	-	-
	*l* (Å)	2.167	2.129	-	-

**Figure 1:**
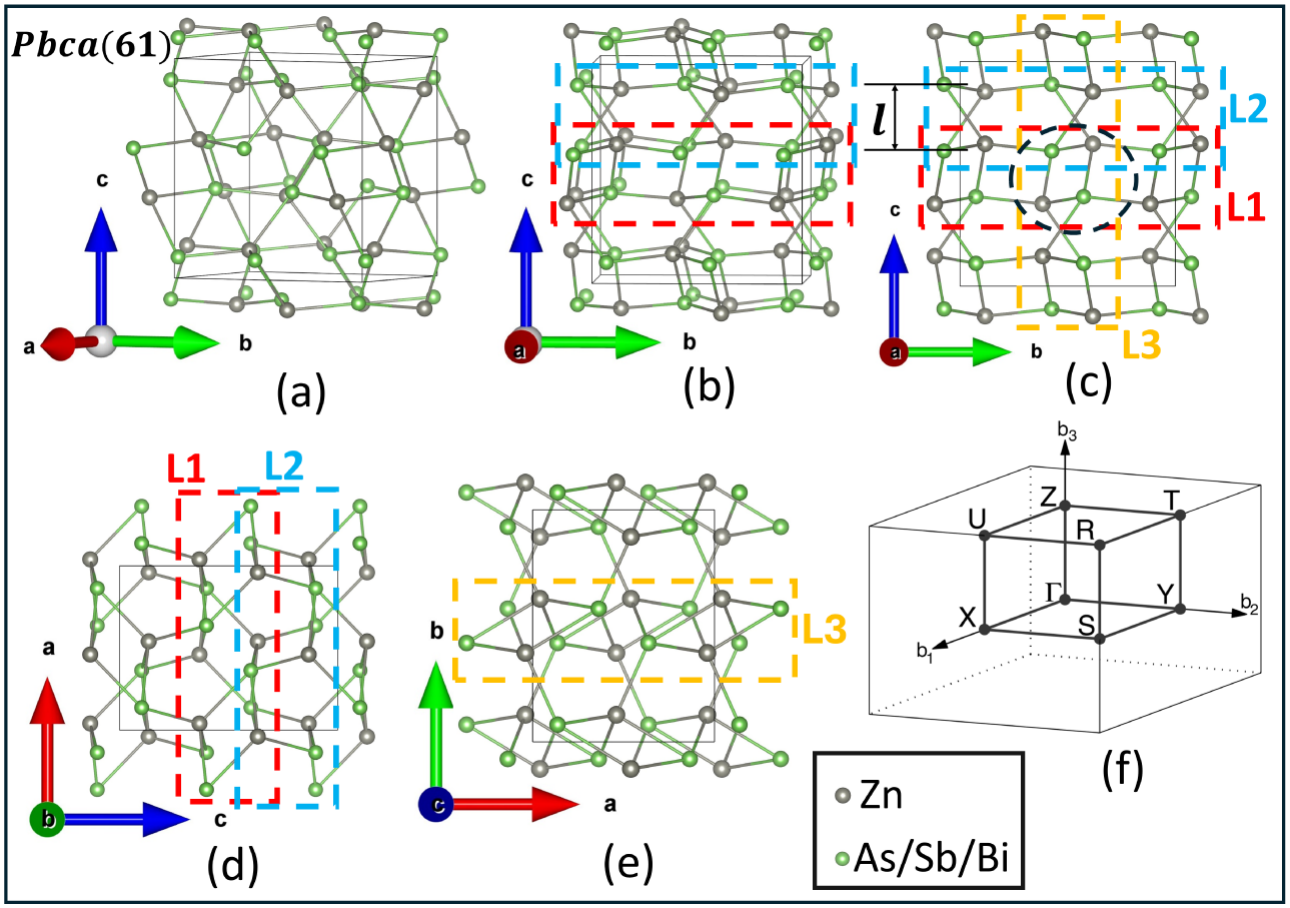
Schematic diagram of the 3D bulk structure of ZnX (X= As, Sb, and Bi) with space group *Pbca*(*No*.61) showing (a) the geometrical structure along the (110) plane, slightly rotated along the *ac* plane invariant to the *b*-axis; (b) geometrical structure along the (100) plane, slightly rotated along the *ab* plane invariant to *c*-axis; side views along (c) the (100) and (d) (010) planes, showing possible 2D-(L1 and L2) monolayer structures indicated by red and blue dashed rectangular shapes, respectively. Here, the black dotted circle represents the rhomboid ring associated with the 2D-L1 layer, and *l* is the interlayer separation of L1-like layers (rhomboid layers); (e) top view along the (001) plane showing the possible 2D-L3 monolayer, as indicated by orange color. (f) First Brillouin zone of the orthorhombic lattice showing high-symmetry k-points.

#### Calculation of Formation Energy (Δ*E*)

In this section, we have reported the formation energies (Δ*E*) of the 3D bulk ZnX (X = As, Sb, and Bi) calculated using the general eq 3.^36,55^

3 $$\Delta E\left(\mathrm{ZnX}\right)=E\left(\mathrm{ZnX}\right)-\frac{1}{N_{\mathrm{tot}}}\left[N_{\mathrm{Zn}}E\left(\mathrm{Zn}\right)+N_{X}E\left(X\right)\right]$$

where *E*(ZnX), *E*(Zn), and *E*(X) are the calculated equilibrium energies in eV/atom of the corresponding bulk phases of binary ZnX (X = As, Sb, and Bi) compounds, where the most stable hexagonal structure of Zn belongs to *P*6_3_/*mmc*(*No*.194), and trigonal As, Sb, and Bi belong to *R*3̅*m*(*No*.166) space groups.^56^ Here, *N*_Zn_ is the number of Zn atoms, *N*_X_ represents the number of pnictogen atoms (As, Sb, and Bi), and *N*_tot_ is the total number of atoms that include both Zn and X in the structure of the ZnX unit cell. The computed values of the formation energy (Δ*E*) using PBE (HSE06) functionals for ZnAs, ZnSb, and ZnBi are −0.130 (−0.212), −0.031 (−0.115), and 0.101 (0.028) eV/atom, respectively. The values of Δ*E* using the PBE + SOC method corresponding to ZnAs, ZnSb, and ZnBi are found to be −0.129, −0.027, and 0.132 eV/atom, respectively. Based on the literature, the experimental values of the formation energy for ZnSb range from −0.07 to −0.09 eV/atom. Our calculated value of the formation energy for ZnSb is slightly higher using PBE and PBE + SOC; however, it is in close agreement with the calculated value of Jund et al. (approximately −0.035 eV/atom) using DFT-GGA.^55^ The calculated value of Δ*E* using HSE06 for bulk ZnSb is consistent with experimental results compared to that for the PBE functional. Further, the negative value of Δ*E* suggests that the formation reaction of ZnAs and ZnSb is exothermic, while it is slightly endothermic for ZnBi regardless of the functionals due to its positive value of formation energy. A positive formation energy for ZnBi implies that the compound is not energetically favorable at 0 K and 0 GPa. However, a slight positive formation energy in ZnBi still indicates some possibility of its synthesis through kinetic control, entropic effects, rapid quenching, or the application of specific synthesis conditions despite its unfavorable thermodynamics.^57^

#### Electronic Properties

The electronic properties of the bulk ZnX structures were determined by calculating the charge density difference (Δρ), the atomic and orbital-projected density of states (DOS), and electronic band structures. Here, Δρ is given by the mathematical expression, Δρ = ρ_ZnX_ – ρ_Zn_ – ρ_X_. Necessarily, the values of charge densities of noninteracting components, ρ_Zn_ and ρ_X_ are computed considering a fixed geometry or lattice parameters of the interacting system, from which ρ_ZnX_ is calculated.

Qualitatively, the 2D contour plots of charge density difference elucidate the nature of covalent bonding in ZnX in terms of the Zintl–Klemm principle, where Zn^2+^ acts as an electron donor and X^2–^ acts as an electron acceptor. X^2–^ itself does not satisfy the octet electronic configuration, so it is covalently bonded to another X^2–^ forming a X_2_^4–^dimer. Figure 2 represents the 2D contour plot of charge density difference (Δρ) along the slice plane passing through the plane of the rhomboid ring, where the Zn–Zn bond forms the shortest diagonal. Here, the color scale bar represents charge depletion (red) and charge accumulation (blue). Since the maximum value of Δρ is at the center of the interatomic line of ZnX, shifting slightly toward the electronegative X atom, indicating higher bond polarity. Also, the Δρ value is localized more for ZnX with a smaller unit cell volume, where the sides Zn–X of the planar rhomboid exist in shorter bond lengths. It can be speculated from the distribution of Δρ that there exists a covalently bonded multicenter framework. While viewing from the top of the rhomboid plane, the maximum value of Δρ is localized toward the X atom, forming the vertex of a triangle.

**Figure 2:**
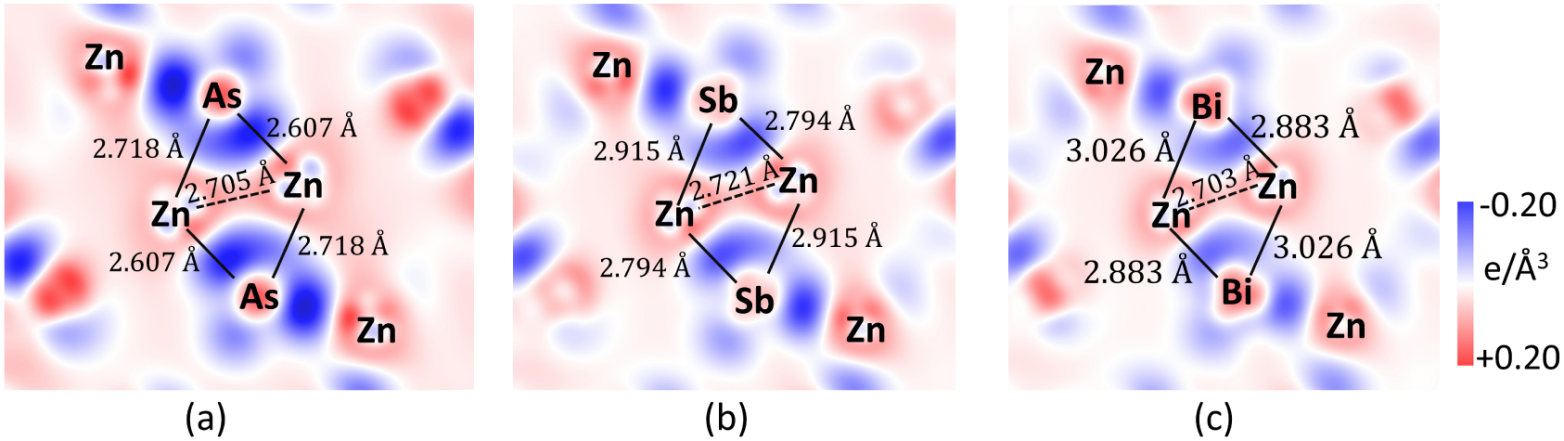
Diagram representing the 2D contour plot of charge density difference (Δρ) with a slice plane passing through the plane of the rhomboid ring Zn_2_X_2_ in 3D bulk structures of (a) ZnAs, (b) ZnSb, and (c) ZnBi. The red and blue colors in the color scale bar represent charge depletion and augmentation, respectively. The numbers in the inset represent the bond lengths constituting the sides of the rhomboid ring.

In addition, the total and partial (atomic and orbital projected) DOS and electronic band structures reveal a clear picture of the electronic properties of the 3D bulk from which it can be confirmed that bulk ZnAs and ZnSb are narrow band gap semiconductors, while bulk ZnBi is a semimetal, as determined using both PBE and HSE06 functionals, as shown in Figures 3 and 4. From the atom-projected DOS, we observe that the electronegative atom X has more contributions near the Fermi level. In bulk ZnAs, Zn-s,p and As-s,p contribute more at the conduction band minimum (CBM), with a major contribution from the As atom. Similarly, As-p and Zn-s,p contribute more at the valence band maximum (VBM). In the case of ZnSb, Zn-p and Sb-p contribute more at the VBM, while Zn-s,p and Sb-s,p contribute more at the CBM; however, in the case of bulk ZnBi, Bi-p and Zn-s,p have major contributions at the Fermi level (*E*_F_). Consequently, the significant d–p hybridization between Zn-3d and X-5p orbitals in the vicinity of the Fermi level (*E*_F_ − 2.0 eV to *E*_F_ eV) implies strong covalent bonding between Zn and X atoms. Additionally, the valence s electrons of X are localized well below the Fermi level, whereas Zn has its valence s electrons at the valence band edge nearest to the Fermi level in ZnAs; however, in bulk ZnBi, the partially filled s orbitals are localized at the Fermi level, which causes a greater tendency for Zn to form tetrahedral coordination in the structure compared to X atoms. From the electronic band structure calculated using the high symmetry k-path corresponding to the first Brillouin zone (BZ), as shown in Figure 1f, we observe that bulk ZnAs and ZnSb exhibit a narrow and indirect band gap of 0.28 (0.97) eV and 0.02 (0.61) eV, respectively, using the PBE (HSE06) functional. The band gap slightly reduces to 0.27 eV in ZnAs, whereas the gap closes in ZnSb with the inclusion of SOC due to the VBM and CBM just touching the Fermi level. There is no band gap opening in bulk ZnBi using both the PBE and HSE06 functionals at an ambient pressure of 0.0 GPa and a temperature of 0.0 K. The band structure is characterized by multivalley features in the conduction band in ZnAs and ZnSb. In addition, there exists distinct hole and electron pockets at different regions of the Brillouin zone in ZnBi along the high-symmetry k-path Γ–*X* and Γ–*Z,* as shown in Figure 4 indicating its semimetallic property. Our calculated value of the band gap, 0.02 eV, in bulk ZnSb using GGA-PBE is too small in comparison to the experimental value of 0.50 eV; however, the calculated value of the band gap for ZnSb using HSE06 is consistent with the experimental results. It is a well-known fact that the standard PBE significantly underestimates the band gap. This band gap value well agrees with 0.03 eV and 0.05 eV obtained by Jund et al.^55^ and Benson et al.,^23^ respectively, calculated using the same PBE parametrization. Furthermore, our band gap value for bulk ZnSb using the HSE06 functional is in close agreement with that of 0.56 eV calculated by Niedziolka, Jund et al. using the same hybrid functional.^21,58^ Similarly, it is also in agreement with the value of 0.60 eV obtained by Yamada in 1978 using an empirical pseudopotential method (EPM) developed by Cohen and Heine.^59,60^ Similarly, our HSE06-calculated value of the electronic band gap for 3D bulk ZnAs agrees with the experimental value of 0.90 eV. However, the PBE-calculated value though underestimated still agrees with the other calculated value of 0.30 eV using the same PBE parametrization.^23^ The electronic band gap for ZnAs, ZnSb, and ZnBi with PBE + vdW attains values of 0.29, 0.02, and 0.00 eV, respectively, which remain almost the same as the PBE-calculated values w/o vdW interaction. The total electronic band structures for ZnAs, ZnSb, and ZnBi, considering van der Waals inetraction in combination with the PBE functional are shown in Figure S1.

**Figure 3:**
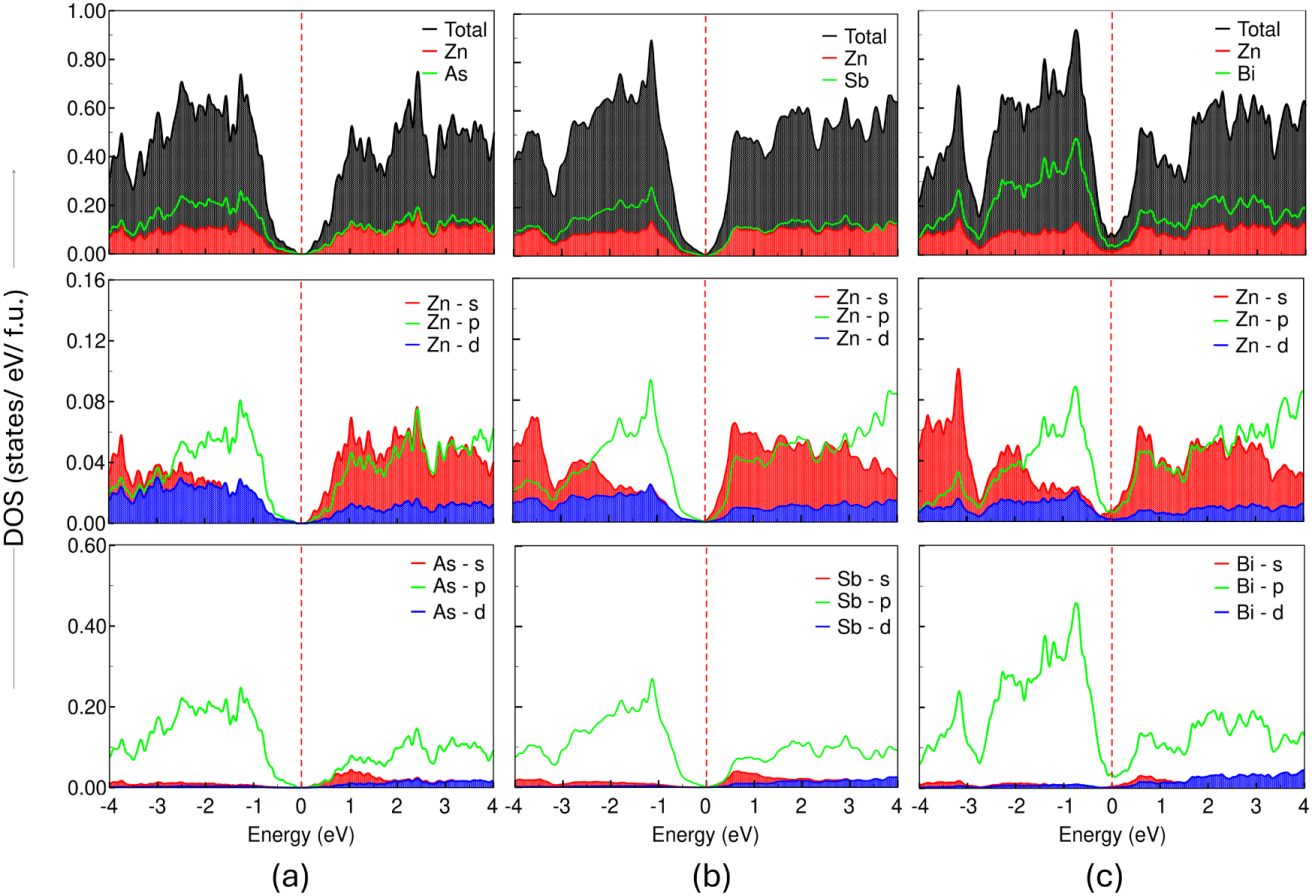
Total DOS, atomic, and orbital-projected DOS for 3D bulk ZnX, (a) ZnAs, (b) ZnSb, and (c) ZnBi, calculated at *T* = 0.0 K using the PBE functional. In all the plots, the zero of the energy axis represents the Fermi level (*E*_F_), which is indicated by a dashed vertical red line.

**Figure 4:**
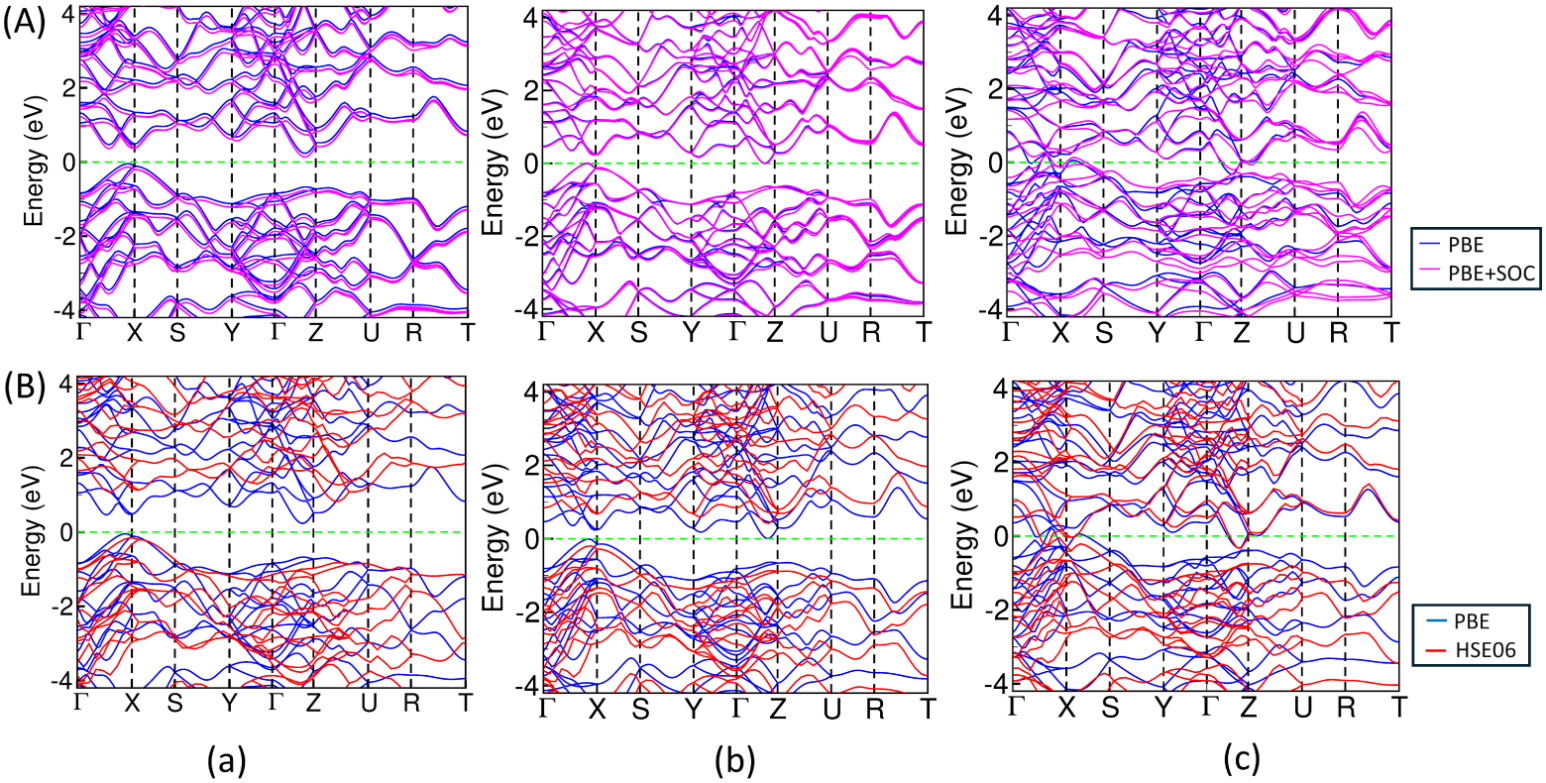
Diagram representing comparative electronic band structures using (A) PBE and PBE + SOC and (B) PBE and HSE06 functionals for the 3D bulk ZnX, (a) ZnAs, (b) ZnSb, and (c) ZnBi. The zero of the energy axis represents the Fermi level (*E*_F_), which is indicated by a dashed horizontal green line.

### Crystal Symmetry in 2D-ML ZnX Derived from 3D Bulk

The feasibility of two-dimensional monolayer (2D-ML) structures derived from the orthorhombic phase of 3D bulk ZnX with a *Pbca* space group has been studied via crystal modeling of three different monolayers, L1, L2, and L3, highlighted by red, blue, and orange dashed rectangular shapes, respectively (Figures 1 and 8). The unit cell of each 2D-(L1, L2, and L3) monolayers is composed of 4 Zn and 4 X (As, Sb, and Bi) atoms. The computational model indicating relaxed geometrical structure of the primitive unit cell of the 2D-L1 monolayer with a vacuum thickness of ∼30 Å is shown in Figure S2. In this study, we prefer to fully relax all the lattice vectors of the 2D monolayers together with atom positions to get an accurate description of the crystal symmetry and lattice parameters.^61^ The 2D monolayers L1, L2, and L3 are constructed via lattice engineering, following the dimensional reduction of the 3D bulk structure. The fully relaxed geometry of the 2D-(L1, L2, and L3) monolayers exhibits the crystal symmetry of a rectangular lattice characterized by *a*  ≠  *b*, and γ = 90°. In all three 2D-ML structures, the lattice vectors forming the 2D plane are orthogonal; however, there is slight symmetry breaking along the nonlattice vector *c*-direction in the 2D-L1 and 2D-L3 monolayers. Conversely, a multilayer stacking pattern of our proposed 2D-L1 and 2D-L3 monolayers has not been ascertained as in tetragonal and hexagonal structures.

### Structural Stability in 2D-ML ZnX

We perform detailed investigations on the structural stability of the two-dimensional monolayers ZnX of six different geometries. The three 2D monolayers denoted by 2D-L1, 2D-L2, and 2D-L3 are extracted from the 3D bulk structure of the *Pbca* space group. The remaining three 2D monolayers are extracted from the 3D bulks that belong to the tetragonal symmetry with space group *P*4/*nmm*(*No*.129) of FeSe type, hexagonal (planar honeycomb) symmetry with space group *P6̅m*2­(No.187) of h-BN type, and wurtzite (puckered honeycomb) or trigonal symmetry with space group *P*3*m*1­(*No*.156) of boron phosphide (BP) type. The optimized lattice parameters (*a* and *b*) and the axial angle (γ) for the six different configurations of 2D ZnX are shown in Table 2. From the dimensional analysis, it has been ensured that the 2D-ML structures after the full geometrical relaxation constitute a single 2D cluster, where the two atoms are considered to belong to the same cluster if their distance is less than some scaling of the sum of their covalent radii, given by *d* < *k*(*r*_Zn_ + *r*_X_), where *r*_Zn_ and *r*_X_ are the covalent radii of Zn and X atoms, and *k* = 1.35 represents the scaling factor.^62^

**Table 2:** Optimized Lattice Parameters (*a*, *b*), Axial Angle (γ), and Axial Ratio (*b*/*a*) for the Six Different Configurations of the 2D monolayer of ZnX (X = As, Sb, and Bi) Using the PBE Functional

2D-ZnX	parameters	2D-L1	2D-L2	2D-L3	Tetragonal	Hexagonal	Wurtzite
2D-ZnSb	*a* (Å)	4.551	4.041	5.274	3.816	4.482	4.071
	*b* (Å)	8.815	11.349	7.447	3.816	4.482	4.071
	*b*/*a*	1.94	2.81	1.41	1.00	1.00	1.00
	γ (°)	90	90	90	90	120	120
2D-ZnBi	*a* (Å)	4.633	4.877	5.169	3.861	4.634	4.164
	*b* (Å)	8.885	10.593	7.802	3.861	4.634	4.164
	*b*/*a*	1.92	2.17	1.51	1.00	1.00	1.00
	γ(°)	90	90	90	90	120	120
2D-ZnAs	*a* (Å)	4.647	4.167	5.223	3.693	4.155	4.135
	*b* (Å)	8.134	7.304	6.952	3.693	4.155	4.135
	*b*/*a*	1.75	1.75	1.33	1.00	1.00	1.00
	γ (°)	90	90	90	90	120	120

The relaxed 2D geometrical structure derived from 3D bulk with tetragonal symmetry attains a square lattice (*a* = *b*, and γ = 90°), whereas the 2D lattice symmetry from hexagonal and wurtzite remains unchanged from their bulk counterparts. The geometrical structures of L2 and L3, together with tetragonal, hexagonal, and wurtzite geometries in 2D-ZnX are shown in Figure S3. The energy values calculated using the PBE functional reveal the fact that the 2D-L1 (rectangular) monolayer in ZnAs is the energetically most stable monolayer out of six different structures. The 2D monolayer structure in ZnSb is energetically favorable in both of its 2D-L1, and tetragonal (square) geometries because of their comparable lattice energies, −2.514 and −2.516 eV/atom, respectively, while the 2D monolayer in ZnBi energetically favors tetragonal symmetry. However, the 2D monolayers of ZnX in the planar honeycomb with hexagonal and puckered honeycomb with wurtzite structures are energetically not favorable to a substantial degree compared to the 2D-L1, 2D-L3, and tetragonal structures. The crystal morphology of 2D-L1, 2D-L3, tetragonal, and hexagonal remains similar in ZnAs, ZnSb, and ZnBi, while the relaxed structure of the 2D-L2 monolayer exhibits a different morphology in ZnX, as the two Zn planes move toward each other to spatially lie in a single plane while going from ZnAs to ZnBi, as shown in Figure S3A. Also, the wurtzite geometry in 2D-ZnAs has not been achieved in our DFT calculations, as it converges into a planar honeycomb after full lattice relaxations. However, from a thermodynamic point of view, our study supports the fact that 2D monolayers in ZnX do not exist in hexagonal (planar honeycomb) and wurtzite (puckered honeycomb) geometries; however, they tend to achieve stability in rectangular and tetragonal structures with an orthogonal Bravais lattice system. The numerical values of relative energy per atom in six different configurations of 2D-ML ZnX structures are represented in Figure 5.

**Figure 5:**
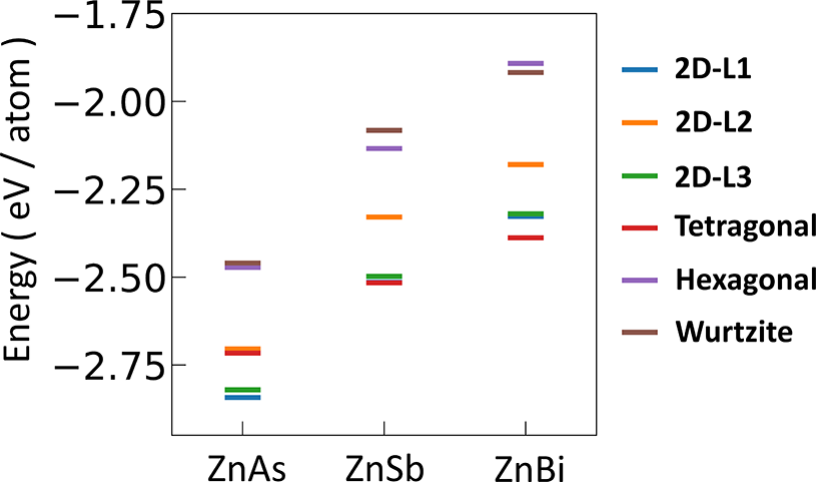
Relative energy (eV) per atom of 2D monolayers in six different structural geometries in ZnX, calculated using the PBE functional. Here, 2D-L1, 2D-L2, and 2D-L3 represent possible monolayers with rectangular symmetry extracted from orthorhombic 3D bulk ZnX (*Pbca* space group).

### Dynamical Stability in 3D Bulk and 2D-ML Structures

The phonon dispersion curves (PHDC) and phonon density of states (PHDOS) are computed using the PBE functional to validate the dynamical stability of the 3D bulk, and their corresponding 2D-L1 monolayer are shown in Figure 6. The PHDCs for the energetically competing structures, 2D-L2, 2D-L3, and tetragonal (square) geometry, are also shown in Figure 7. Also, the PHDC curves for hexagonal and wurtzite symmetries for 2D ZnSb are shown in Figure S4. PHDCs are plotted along high-symmetry points of irreducible Brillouin zone (BZ) boundaries using the phonopy code based on density functional perturbation theory (DFPT).^47^ The DFPT approach explicitly calculates the second-order derivative of the energy and interatomic harmonic force constants. The distribution of acoustic and optical modes in 3D bulk ZnAs and ZnSb agrees with previously calculated results, which validate our calculations for bulk ZnBi and the corresponding 2D structures. The computed vibrational frequencies throughout the Brillouin zone in all the 3D bulk and 2D-L1 monolayers do not show any imaginary components, indicating their dynamical stability in ZnX, whereas the imaginary frequencies in 2D-L2 and tetragonal lattices in all the pnictide compounds indicate their dynamical instability. Further, the 2D-L3 monolayer in ZnAs is found to be dynamically stable, whereas it is slightly unstable in ZnSb and ZnBi due to the presence of very small negative frequencies around the Γ point. In both the 3D bulk and 2D-L1 monolayers of ZnAs, Zn has a higher contribution in the lower frequency range compared to As, whereas As has a higher contribution in the higher frequency range. However, in ZnSb and ZnBi, the trend of the atomic contribution in phonon modes is reversed compared to that of ZnAs. Herein, we observe distinct imaginary frequencies in some of the 2D-ML models, indicating their instability. The presence of soft modes in phonon dispersion curves suggests that the atomic configuration is susceptible to further distortion along the corresponding vibrational mode to achieve crystal stability. Significant negative frequencies, especially those away from the very lowest acoustic modes, such as in 2D-L2, hexagonal, and wurtzite geometries, almost always indicate a real instability in the structure. However, a small negative frequency close to zero such as in 2D-L3 and tetragonal structures of ZnSb and ZnBi often indicates numerical inaccuracies and insufficient supercell size, which can be improved using advanced computational resources. If the small negative frequencies are not due to computational limitations, lattice stability can be enhanced, getting rid of these small negative frequencies by applying a small amount of strain along the lattice vectors.^63^ Therefore, our present calculated results suggest that the 2D-L1 monolayer retrieved from the 3D bulk geometry is found to be energetically and dynamically stable at zero strain in all of the ZnX systems, which demands further theoretical and experimental verifications.

**Figure 6:**
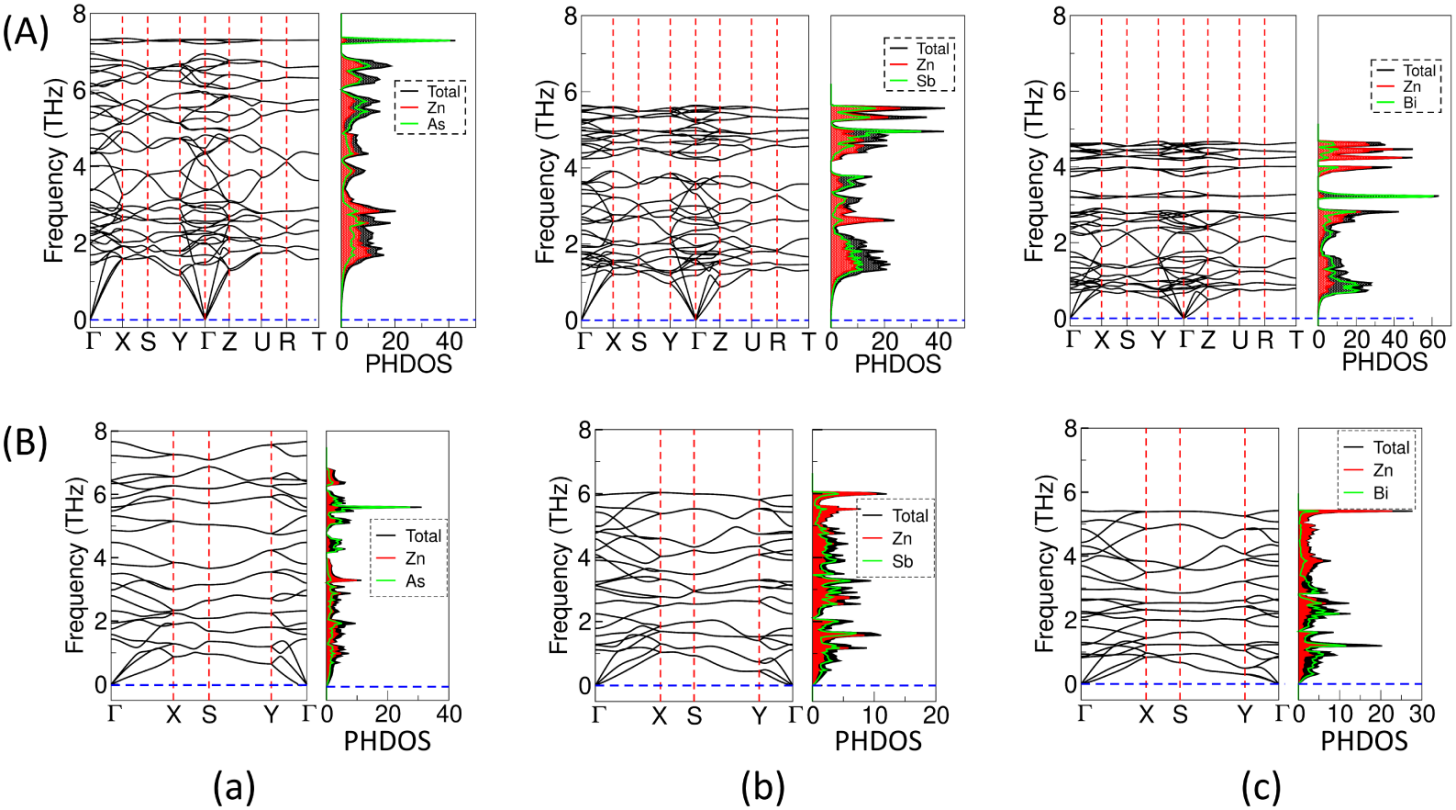
Phonon dispersion curves (PHDC) in THz and phonon density of states (PHDOS) in states/THz, along high-symmetry k-points in (A) 3D bulk and (B) 2D-L1 monolayers of (a) ZnAs, (b) ZnSb, and (c) ZnBi using the PBE functional.

**Figure 7:**
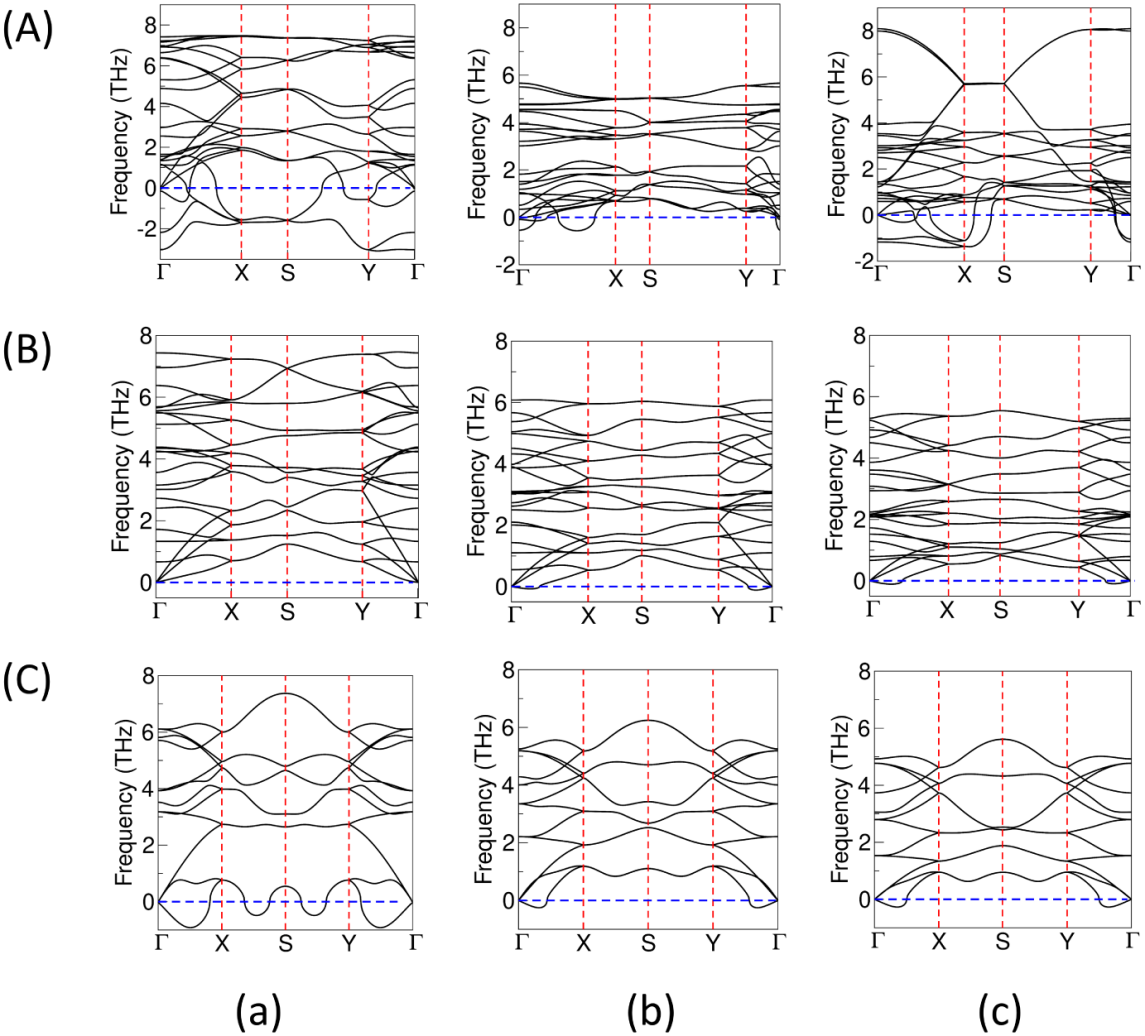
Phonon dispersion curves (PHDCs) in (A) 2D-L2, (B) 2D-L3, and (C) 2D tetragonal monolayers in (a) ZnAs, (b) ZnSb, and (c) ZnBi.

### Calculation of Exfoliation Energy (*E*_exf_)

Exfoliation energy (*E*_exf_) is the energy required to separate an atomic layer (or multiple layers) from the surface of a bulk layered material. Essentially, it also measures the energy needed to overcome the interlayer forces holding the layers together and provides a synthesis route for 2D materials. To facilitate the calculation of exfoliation energy, the self-consistent energies of 2D-(L1, L2, and L3) are calculated after full geometrical relaxation. The order of calculated lattice energy (Δ*E*) per atom in the three different 2D monolayers is Δ*E*(L1) < Δ*E*(L3) < Δ*E*(L2). This trend is consistent across all of the 2D-ML ZnX. Our calculated relative energy values suggest that 2D-L1 is the most stable monolayer compared to L2 and L3 from a thermodynamic point of view. Here, *E*_exf_ is computed for the most stable 2D-L1 structures from the 3D bulk in ZnX, following the procedures explained in the paper^64^ as given by eq 4.

4 $$E_{\mathrm{exf}}=E_{2D-ML}-E_{3D-Bulk}$$

where *E*_2D–ML_ and *E*_3D–Bulk_ are the energy per atom of the 2D monolayer and the 3D bulk, respectively. The calculated exfoliation energy (*E*_exf_) per atom for the 2D-L1 monolayer is 177.65 (210.93), 139.00 (176.29), and 64.00 (66.28) meV/atom for ZnAs, ZnSb, and ZnBi, respectively, using the PBE (HSE06) functionals. The *E*_exf_ values within the PBE + SOC method are 177.72, 138.267, and 59.67 meV/atom for ZnAs, ZnSb, and ZnBi, respectively. There are no noticeable differences in the PBE-calculated values of *E*_exf_ within the inclusion of SOC and without the incorporation of the SOC effect. There are several arguments in the literature regarding the stability of crystal geometry on the basis of exfoliation energy criterion. Ashton et al. suggested that the most promising 2D material candidates will have much lower exfoliation energies of less than 150 meV/atom to be feasible for synthesis. For example, *E*_exf_ for 2D layered vdW materials, graphite (C) and hexagonal boron nitride (h-BN) are found to be 66 and 59 meV/atom, respectively. However, the exfoliation energy of bismene (Bi) is 273 meV/atom, similar to that of 2D antimonene (Sb), which is 236 meV/atom. Both are above the 150 meV/atom threshold for exfoliation energies, yet are experimentally synthesized 2D materials.^65^ Choudhary et al. reported that the conventional energy criterion to predict the feasibility of exfoliation via the computational method is less than 200 meV/atom. Though the energy criterion to predict the feasibility of exfoliation should be less than 200 meV/atom, some of the 2D materials that can be stabilized on suitable substrates such as silicene (Si) and germanium oxide (GeO) monolayers exceptionally take *E*_exf_ values as high as 600 meV/atom.^66^ Based on reports from the available literature, our calculated *E*_exf_ values in 2D-L1 ZnX for ZnAs and ZnSb are relatively higher but still within the acceptable limit of the exfoliation energy criterion, suggesting possibilities for their experimental fabrication. Further, ZnBi in the 2D-L1 structure represents a promising 2D material because of its extremely low *E*_exf_ comparable to that of graphene and h-BN, though the synthesis of its bulk counterpart can be imagined through an endothermic process.

### Ground-State Observables in 2D-L1 Monolayer

#### Structural, Bonding, and Electronic Properties

We establish the possible feasibility of a 2D-L1 monolayer with quasi-diatomic thickness in binary compounds from the 3D orthorhombic ZnX (X = As, Sb, Bi) based on the exfoliation energy criterion. We further analyze the structural geometry, bonding, and electronic properties of this 2D monolayer. There exists significant reorganization of the atom positions locally owing to the effect of dimensional reduction in these 2D-L1 monolayers. Changes in the lattice vectors occur after full optimization, leading to rectangular symmetry with orthogonal Bravais lattice vectors. Further, in this monolayer, four Zn and four X atoms occupy the Wyckoff positions of 4e and 4e, respectively, which constitute a chain of Zn_2_X_2_ rhomboid rings connected through the X–X dimers along the *ab* plane as shown in Figure 8c–e. Here, the 2D-L1 monolayer is characterized by an undulating planar surface with X atoms periodically buckled upward and downward with respect to the Zn plane when viewed along (100). In addition, there exist two Zn planes along the [001] direction, which are slightly offset from each other with their vertical separation of 0.60 Å, 0.02 Å, and 0.06 Å in ZnAs, ZnSb, and ZnBi, respectively, representing their quasi-diatomic thickness instead of a planar monatomic layer. The calculated values of the bond lengths (*w*, *x*, *y*, *z*) and the buckling height *h*_1_ and *h*_2_ along the rhomboid chain in the 2D-L1 monolayer of ZnX are represented in Table 3. Here, *h* = *h*_1_ + h_2_ represents the total thickness of the monolayer along the *c* direction.

**Figure 8:**
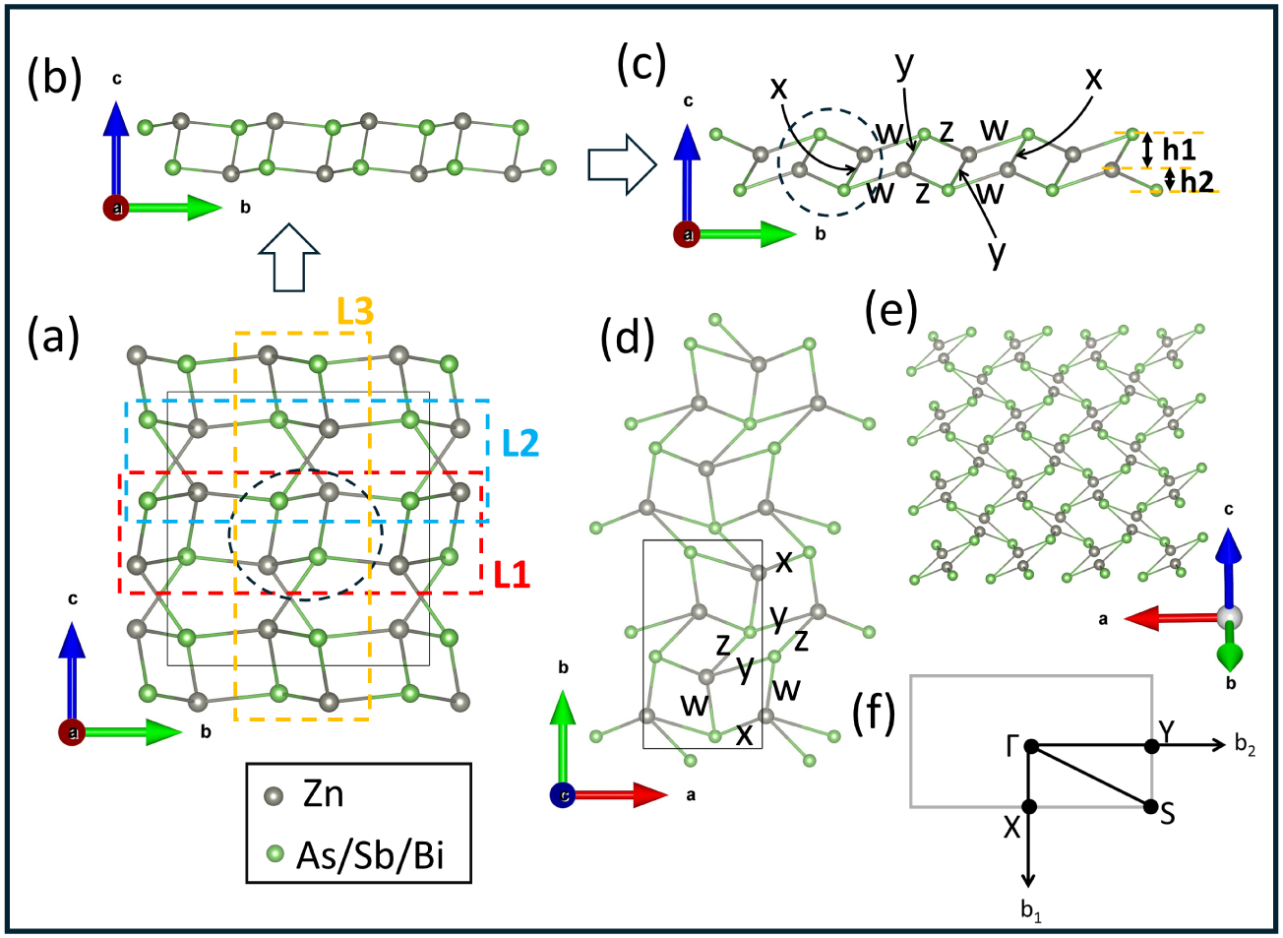
Schematic diagram of (a) the 3D bulk structure of ZnX (X= As, Sb, and Bi), showing the three possible 2D-ML structures, L1, L2, and L3, indicated by red, blue, and orange dashed rectangular boxes, respectively, along the (100) plane. The black dashed circle represents the rhomboid ring. (b) Unrelaxed geometrical structure of the energetically favored 2D-L1 monolayer in ZnX viewed along the (100) plane. (c)–(e) Relaxed geometrical structure of 2D-L1, along different crystallographic planes (100), (001), and (1̅1̅0), respectively. Here, *h*_1_ and *h*_2_ represent the buckling height of the X (As/Sb/Bi) atoms buckled upward and downward with reference to the Zn plane along the *c*-axis. (f) First Brillouin zone of the rectangular 2D lattice.

**Table 3:** Calculated Bond Lengths (*w*, *x*, *y*, *z*), the Buckling Height *h*_1_, and *h*_2_ of X (As, Sb, and Bi) Atom with Respect to the Zn Plane Corresponding to Figure 8 in the 2D-L1 Monolayer of ZnX Using PBE Functional

parameter (Å)	ZnAs	ZnSb	ZnBi
*w*	2.479	2.767	2.851
*x*	2.561	2.730	2.812
*y*	3.111	2.813	2.909
*z*	2.562	2.795	2.929
*h*_1_	0.796	1.345	1.550
*h*_2_	1.399	1.328	1.437
*h*	2.195	2.673	2.987

From the electronic perspective, the atom-projected density of states (DOS) using the PBE functional reveals that the anion X has more contributions in valence band edge, whereas the cation Zn contributes more to the conduction band edge for all 2D-L1 monolayers in ZnX. The orbital-projected DOS represents the largest contribution of Zn-p in the valence band edge in ZnAs, while in ZnSb and ZnBi, Zn-p,d contributes more to the valence band edge, whereas the Zn-s orbital dominates p and d with the least contribution from the Zn-d orbital in the conduction band edge. Similarly, there exists a substantial contribution from the anion X (As, Sb, Bi)-p orbitals in the valence and conduction band edges. Akin to 3D bulk structures, the 2D-L1 monolayer also exhibits d –p hybridization, thereby preserving the covalent character between Zn and X atoms while going from the 3D bulk to the 2D monolayer. The total DOS (Figure 9) and electronic band structures (Figure 10) calculated along the high-symmetry K-path, Γ­(0,0,0)–*X*(0.5,0,0)–*S*(0.5,0.5,0)–*Y*(0,0.5,0)−Γ­(0,0,0) corresponding to the first BZ as shown in Figure 8f, represent the 2D-L1 monolayer of ZnX, as a semiconductor with a wide band gap of 1.52 (2.30) eV in ZnAs, 0.94 (1.71) eV in ZnSb, and 0.63 (1.26) eV in ZnBi, respectively, using PBE (HSE06) functionals. The electronic band structure is slightly affected in 2D ZnSb and ZnBi when using the relativistic SOC method because of the shifting of the conduction band minimum (CBM) toward the Fermi level. The electronic band structures reveal band gap values of 1.52, 0.91, and 0.52 eV in the 2D-L1 monolayer of ZnAs, ZnSb, and ZnBi, respectively, within the inclusion of the SOC effect. The nature of the band gap is slightly indirect in ZnAs, whereas it is direct in ZnSb and ZnBi, irrespective of the exchange functionals and computational methods. Surprisingly, the ZnBi structure undergoes an electronic transition from semimetal to semiconductor while going from the 3D bulk to the 2D monolayer, as manifested in the electronic band structures using PBE and HSE06 functionals.

**Figure 9:**
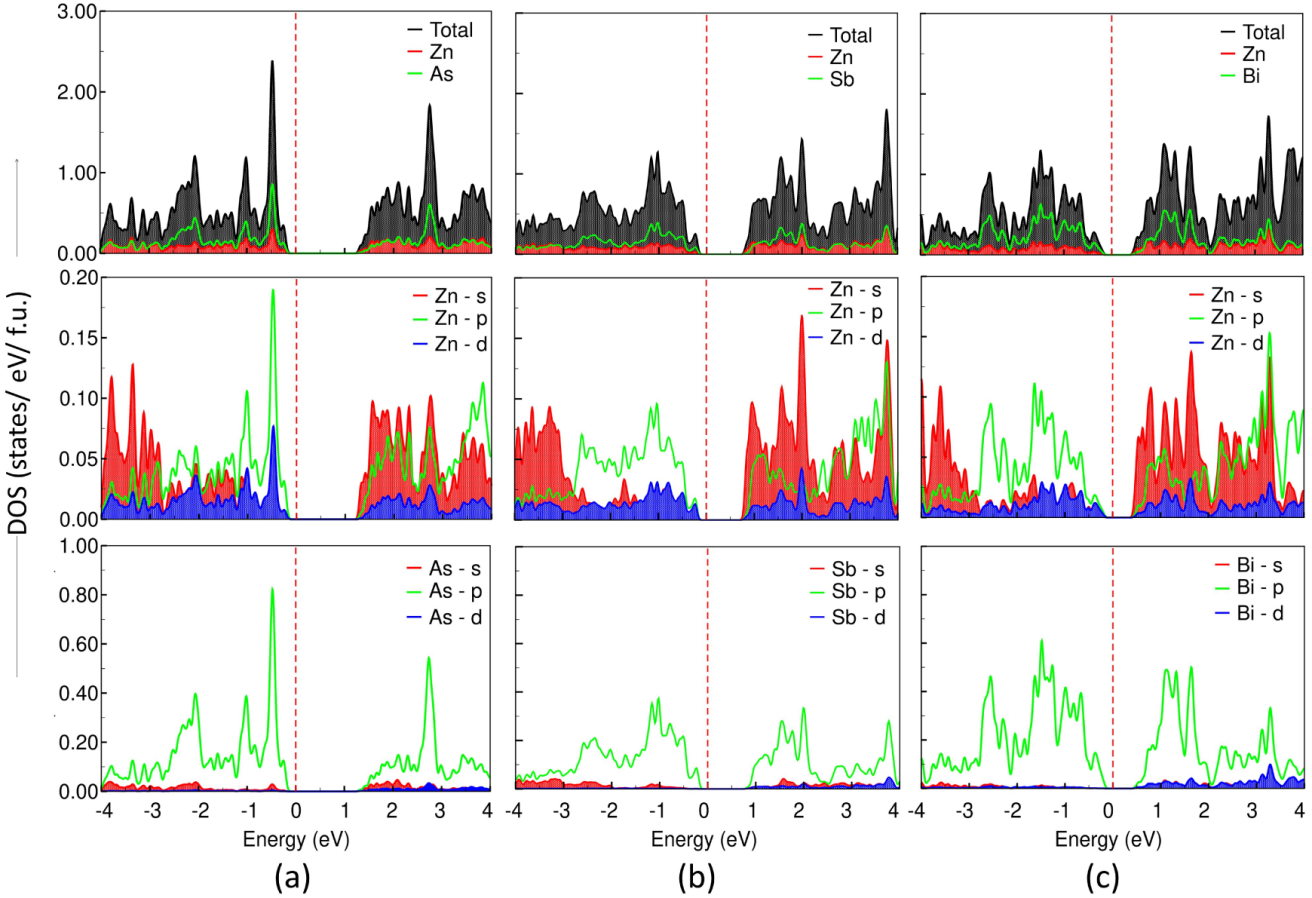
Total DOS, atomic, and orbital-projected DOS for the 2D-L1 monolayer of (a) ZnAs, (b) ZnSb, and (c) ZnBi, calculated at *T* = 0.0 K using the PBE functional. In all the plots, the zero of the energy axis represents the Fermi level (*E*_F_), which is indicated by a dashed vertical red line.

**Figure 10:**
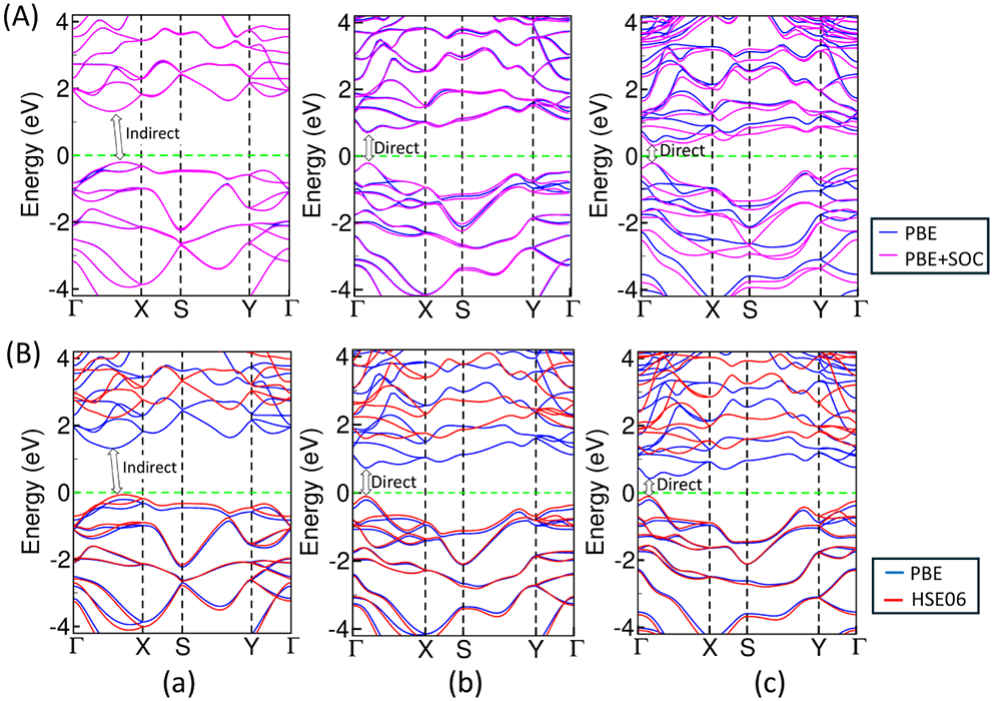
Diagram representing comparative electronic band structures using (A) PBE and PBE + SOC and (B) PBE and HSE06 functionals for the most stable 2D-L1 monolayer in ZnX: (a) ZnAs, (b) ZnSb, and (c) ZnBi. The zero of the energy axis represents the Fermi level (*E*_F_), which is indicated by a dashed horizontal green line.

#### Mechanical Stability

Mechanical stability refers to the resistance of materials toward deformations or distortions in the presence of external strain, with a certain degree of anisotropy. The mechanical stability of the 2D-L1 ZnX has been studied using the energy-strain approach, where the elastic stiffness tensor has been derived from the second-order derivative of the total energies versus strain. The elastic energy Δ*U*[*V*,ϵ_*i*_] of a solid material under strain in the harmonic approximation is given by

5 $$\Delta U\left(V,\epsilon_{i}\right)=U\left(V,\epsilon_{i}\right)-U\left(V_{o},0\right)=\frac{V_{o}}{2}\overset{6}{\underset{i,j=1}{\sum }}C_{ij}\epsilon_{j}\epsilon_{i}$$

where *U*(*V*,ϵ_*i*_) and *U*(*V*_o_,0) are the total energies of the distorted cell with volume *V* and the undistorted cell at equilibrium volume *V*_o_, respectively. For two-dimensional anisotropic materials, the energy–strain relationship is given by,

6 $$\Delta U\left(V,\epsilon_{i}\right)=\frac{V_{o}}{2}\left[\epsilon_{xx}{,\epsilon }_{yy}\;\;\;2\epsilon_{xy}\right]\left[\begin{array}{ccc} C_{11} & C_{12} & C_{16}\\ C_{21} & C_{22} & C_{26}\\ C_{61} & C_{62} & C_{66} \end{array}\right]\left[\begin{array}{c} \epsilon_{xx}\\ \epsilon_{yy}\\ 2\epsilon_{xy} \end{array}\right]$$

where ϵ_*xx*_, and ϵ_*yy*_ are normal strain components and ϵ_*xy*_ is shear strain. There are four independent elastic constants based on the symmetry of the 2D materials under study: *C*_11_, *C*_12_, *C*_22_, and *C*_66_, where *C*_11_ and *C*_22_ represent the stiffness along the *x* and *y* directions, *C*_12_ gives the coupling between strains along the *x* and *y* directions, *C*_16_ and *C*_26_ represent the shear coupling effects, and *C*_66_ gives resistance to shear strain. For the orthotropic 2D-L1 monolayer in ZnX, *C*_16_ = *C*_26_ = 0. In addition, the symmetry condition ensures *C*_12_ = *C*_21_, *C*_16_ = *C*_61_, and *C*_26_ = *C*_62_. Here, we calculate *C*_*ij*_, and the total energy in terms of ϵ in the strain range −2.0% ≤ ϵ ≤ +2.0% with an increase of 0.5%. The calculated values of elastic constants (*C*_*ij*_) and the minimum and maximum values of Poisson’s ratio (ν), using the PBE functional are represented in Table 4. In addition, the Voigt–Reuss–Hill averages of bulk modulus (*B*) and shear modulus (*G*)^67^ for the 2D-L1 monolayer in ZnX are shown in Table S1.

**Table 4:** Calculated Value of Four Independent Elastic Constants *C*_11_, *C*_12_, *C*_22_, and *C*_66_, and the Minimum (Min) and Maximum (Max) Values of Poisson’s Ratio (ν) in the 2D-L1 Monolayer of ZnX (X = As, Sb, Bi) Using the PBE Functional

properties	ZnAs	ZnSb	ZnBi
*C*_11_	11.511	39.125	35.997
*C*_12_	11.649	11.732	6.496
*C*_22_	45.799	24.394	26.567
*C*_66_	12.894	6.049	3.668
ν_min_	–0.058	0.300	0.180
ν_max_	1.012	0.561	0.671

Our calculations reveal the fact that the proposed 2D-L1 monolayer in the ZnX (X= As, Sb, Bi) lattice satisfies sufficient elastic stability conditions, *C*_11_ > 0, *C*_22_ > 0, and *C*_11_ × *C*_22_ > 0, indicating its mechanical stability. Furthermore, the range of Poisson’s ratio (ν) in the 2D-L1 monolayer of ZnAs, −0.058 to 1.012, signifies the auxetic property of this material owing to its possibility of a negative Poisson’s ratio. Generally, materials exhibit a positive Poisson’s ratio, which becomes thinner (or thicker) in the lateral directions when applying longitudinal tension (or compression). In contrast, a few 2D materials such as GaP, AlP, ZnS, and B_4_N, with negative Poisson’s ratios are called auxetic materials that behave counterintuitively, i.e., expand when stretched and contract when compressed. Due to their unprecedented mechanical ability, materials with negative Poisson’s ratios attract considerable attention toward numerous promising applications, particularly in aerospace and defense mechanisms, tissue engineering, bulletproof vests, and personal protective gear enhancement, etc.^68–72^

#### Thermal Stability

The thermal stability of a material represents its ability to resist decomposition, phase changes, and structural integrity under heating or elevated temperatures. In other words, it measures the ability of the locally stable structure to resist thermal motion. It can be determined by checking whether the chemical bonds are reconfigured, broken, or remain roughly unchanged during *ab initio* molecular dynamics (AIMD) simulations over a long time and at elevated temperatures. To verify the thermal stability of the proposed 2D-L1 monolayer, AIMD simulations of a supercell (4 × 4 × 1) with a total of 128 atoms has been employed in the *NVT* ensemble; that is, the number of atoms (*N*), volume (*V*), and temperature (*T*) remain constant throughout the simulation time, where the temperature is controlled by scaling the velocities. The time step and the duration of the time are set to 0.5 and 4500 fs, respectively.^73–75^ We find that the total potential energy of the 2D-L1 monolayer in ZnX remains constant when heated at a room temperature of 300 K without significant changes in the structural geometry, which indicates the thermal stability of the materials. The time evolution graph can confirm the stability of the system and the absence of unexpected phase transitions or degradation under the given conditions. Although there exists some degree of distortion in the bond length due to atomic vibrations, this can be treated as normal due to natural thermal energy fluctuations. Based on our calculated results, we predict that the 2D-L1 monolayer in ZnX candidates is stable at room temperature. The variation in total potential energy with time at 300 K in the 2D-L1 monolayer of ZnX along with snapshot of atomic configurations at the end of the simulation can be observed in Figure 11.

**Figure 11:**
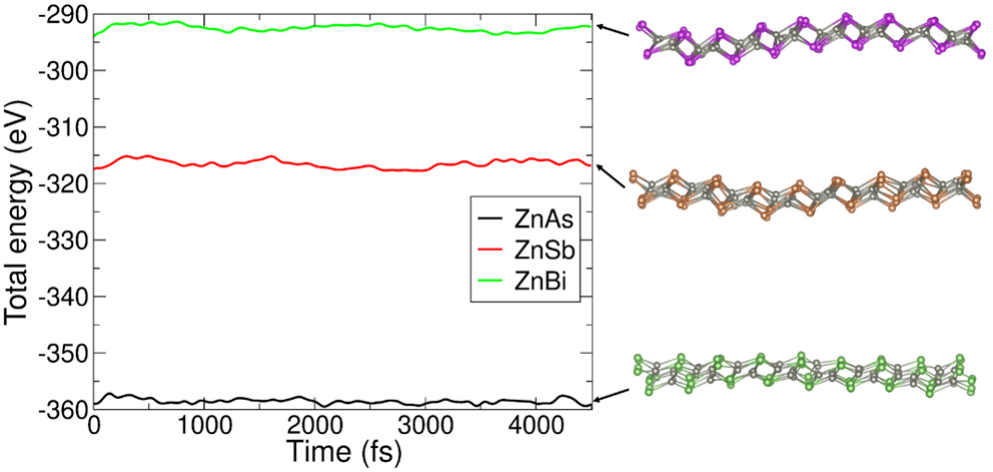
Time-dependent potential energy curves for the 2D-L1 monolayer in ZnX (X = As, Sb, Bi) heated at 300 K with snapshots of their geometrical configurations at 4500 fs. Here, the gray sphere represents the Zn atom; green, brown, and purple represent As, Sb, and Bi atoms, respectively.

### Electronic Transitions in 2D-ML Structures

The electronic band structures of the energetically competing 2D-ML phases that include 2D-L1, 2D-L3, and the 2D tetragonal monolayer in ZnX elucidate the fact that the rectangular 2D-L1 and 2D-L3 monolayers derived from the 3D bulk ZnX with orthorhombic symmetry are wide band gap semiconductors. The band gap tends to be direct while going from ZnAs to ZnBi in the 2D-L1 monolayer, whereas the nature of the band gap is always indirect in the 2D-L3 monolayer, irrespective of the pnictogen atom. The band gap values in 2D-L3 are 1.53, 0.91, and 0.52 eV, corresponding to ZnAs, ZnSb, and ZnBi using the PBE functional. The 2D tetragonal monolayers in ZnX are metallic due to frontier orbitals overlapping at the Fermi level, as shown in Figure 12. The electronic transition from wide band gap semiconductors in 2D-(L1, L3) to the gapless metallic structure in tetragonal geometry in 2D-ML ZnX is an intriguing result of our calculations.

**Figure 12:**
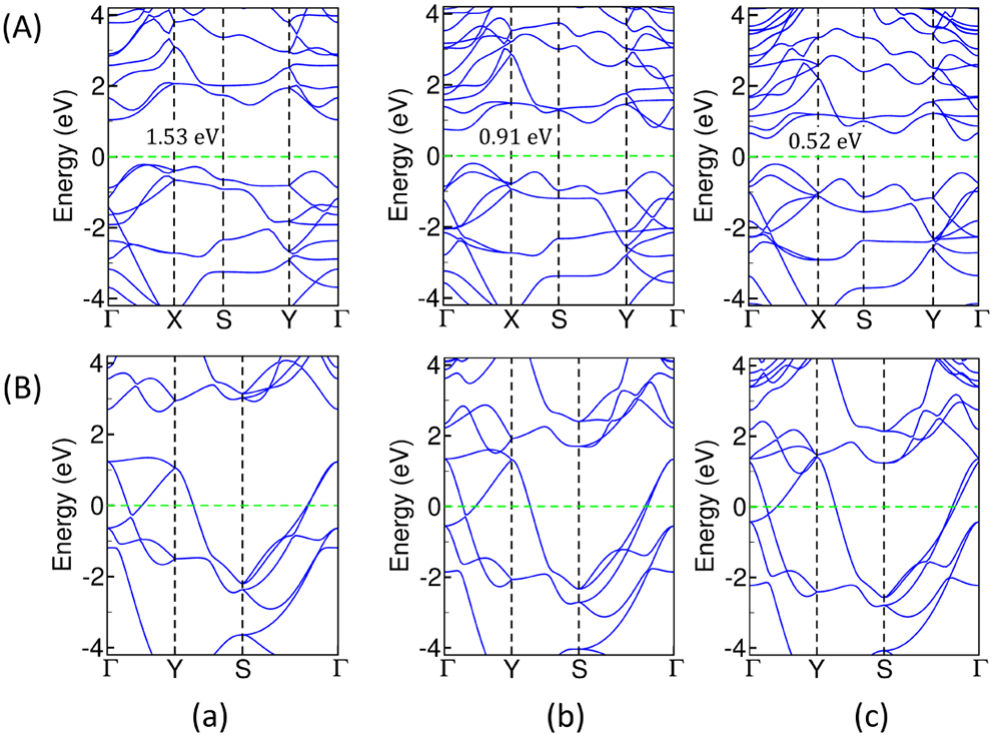
Diagram representing electronic band structures in (A) 2D-L3, where the numbers in the inset represent the band gap values, and (B) 2D tetragonal monolayers using the PBE functional in (a) ZnAs, (b) ZnSb, and (c) ZnBi. The red and black solid bands represent the VBM and CBM in the 2D tetragonal structure. The zero of the energy axis represents the Fermi level (*E*_F_), which is indicated by a dashed horizontal green line.

## Conclusion

In this study, we have performed a comprehensive investigation of the structural, electronic, and thermodynamic stabilities of the 3D bulk ZnX (X= As, Sb, and Bi) in orthorhombic symmetry (space group *Pbca*) and their possible 2D candidates using quantum mechanical DFT and a lattice engineering approach. The thermoelectric 3D bulk ZnSb has been reported to be stable at ambient pressure and temperature in orthorhombic symmetry up to the present date, whereas the experimental synthesis of orthorhombic ZnAs and ZnBi can be realized only at slightly elevated pressure and temperature. However, the dynamical stability of bulk ZnAs and ZnBi has still been manifested via the absence of imaginary frequencies in phonon dispersion curves at 0 K and 0 GPa, suggesting their possible structural stability. The feasibility and stability of the 2D monolayer of such binary compounds have been explored, providing new avenues for predicting and synthesizing the 2D monolayer of ZnX. This study performs a systematic exfoliation of 2D monolayers L1, L2, and L3 along different crystallographic directions, satisfying their exfoliation energy criterion. We compare the structural stability and relative energies of these L1, L2, and L3 monolayers derived from the 3D bulk with those of the tetragonal, hexagonal (planar honeycomb), and wurtzite (puckered honeycomb) structures. Our DFT calculations suggest that the 2D-L1 structure originating from the quasi-layered rhomboid rings in the 3D bulk, is the promising candidate for the 2D monolayer in ZnX, satisfying all the stability criteria from the thermal, mechanical, and dynamical points of view. Further, it also tends to stabilize dynamically in the 2D-L3 structure in ZnAs, though slightly higher in energy by 0.022 eV (using PBE) compared to its 2D-L1 geometry. We also observe that the 2D tetragonal structure in ZnSb and ZnBi energetically competes with 2D-L1; however, the tetragonal monolayer shows the signature of dynamical instability due to small negative frequencies around the Γ point. The electronic band structures and density of states indicate that the 2D-L1 and 2D-L3 monolayers with rectangular symmetry in ZnX are semiconductors with a wide band gap. The nature of the band gap is slightly indirect in 2D-ZnAs, whereas it is direct in 2D-ZnSb and 2D-ZnBi corresponding to the 2D-L1 monolayer. However, it is always indirect in the 2D-L3 monolayer. Interestingly, there exists an electronic transition from a semiconductor in rectangular 2D-L1 to metallic in the 2D tetragonal structure. Owing to the direct nature of 2D-L1 monolayers in ZnSb and ZnBi, the application of such materials can be undoubtedly found in solar cells, integrated circuits, and flexible electronics. Recently, 2D materials with a wide band gap have been found to have profound applications in optoelectronic devices working under blue or ultraviolet (UV) light.^61^ Additionally, the signature of a negative Poisson’s ratio (ν) in the 2D-L1 monolayer indicates an auxetic property in ZnAs that can be utilized in aerospace and automotive engineering, energy storage, protective gear units, shock absorption, etc., requiring further experimental and theoretical investigation of these unprecedented 2D structures.

## Supplementary Material



## Notes

Total electronic band structure plots for 3D bulk structures of (a) ZnAs, (b) ZnSb, and (c) ZnBi with orthorhombic symmetry and space group, *Pbca* with and without (w/o) considering the van der Waals (vdW) interaction; diagram representing a computational model of the primitive unit cell of the most stable 2D-L1 monolayer in ZnX (X= As, Sb, and Bi) with rectangular symmetry; relaxed geometrical structures of 2D-L2, 2D-L3, tetragonal (square), hexagonal (planar honeycomb), and wurtzite (puckered honeycomb) monolayers in ZnX; phonon dispersion curves (PHDCs) in 2D monolayers with (a) hexagonal (planar honeycomb) and (b) wurtzite (puckered honeycomb) structures in ZnSb; table representing Voigt–Reuss–Hill averages of bulk modulus (*K*) and shear modulus (G) in the 2D-L1 monolayer of ZnX calculated using the PBE functional (PDF)
