# Supplementary material for: Lattice Engineering Novel 2D Monolayer in Zinc Pnictides
Source: ACS Omega. 2025 Oct 24;10(43):51088–102. doi: 10.1021/acsomega.5c05775 (PMC12593096; doi:10.1021/acsomega.5c05775)
Supplement: Supplementary file 1 [file ao5c05775_si_001.pdf]

Lattice engineering novel 2D monolayer in zinc pnictides

Dinesh Thapa*,†, and Seong-Gon Kim‡

†Department of Mathematics and Physics, Thomas More University, Crestview Hills, Kentucky 41017, United States
‡Department of Physics and Astronomy, Mississippi State University, Mississippi 39762, United States

*****_To whom correspondence should be addressed:_ ***thapad@thomasmore.edu***

Supplementary Information

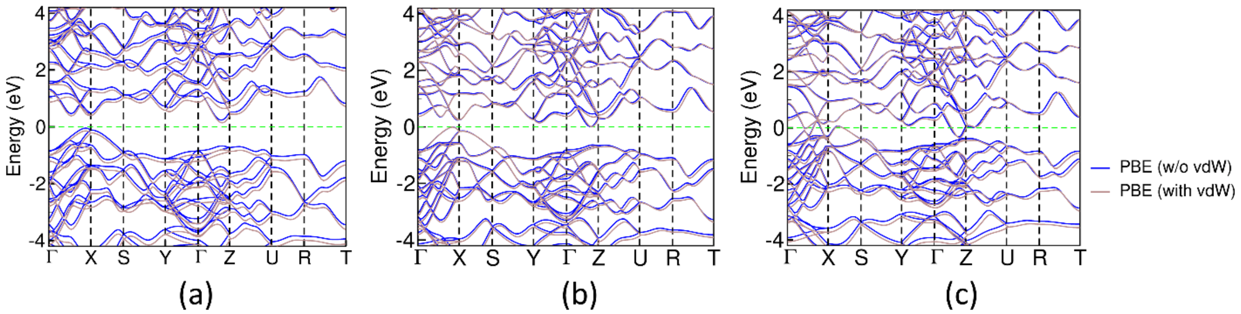

Fig. S1 Total electronic band structure plots for 3D-bulk structures of (a) ZnAs, (b) ZnSb, and (c) ZnBi of orthorhombic symmetry with space group, *Pbca*. The blue and brown solid lines represent total electronic band structure using PBE without (w/o) and with van der Waal (vdW) interaction, respectively. Zero of energy axis represents the Fermi level which is indicated by the green horizontal dashed line.

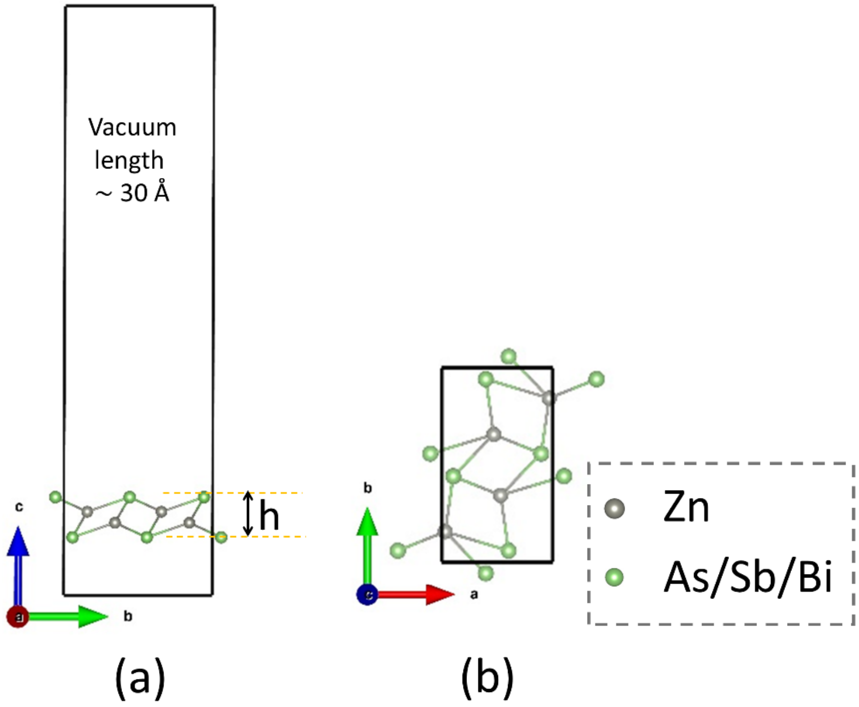

Fig. S2 Diagram representing a computational model of the primitive unit cell of the most stable 2D-L1 monolayer in ZnX (X= As, Sb, and Bi) with rectangular symmetry with (a) side view along (100) plane, and (b) top view along (001) plane. Here, the solid black line represents the primitive unit cell size, and **h** represents the total thickness of the monolayer.

Table S1: Voigt-Reuss-Hill averages of Bulk modulus (K), and Shear modulus (G) in 2D-L1 monolayer of ZnX calculated using PBE functional.

2D-L1 (ZnX)		Voigt	Reuss	Hill
ZnAs	K (N/m)	20.152	11.511	15.832
	G (N/m)	10.698	7.056	8.877
ZnSb	K (N/m)	21.746	20.392	21.069
	G (N/m)	8.032	7.358	7.695
ZnBi	K (N/m)	18.889	18.440	18.665
	G (N/m)	8.031	5.629	6.830

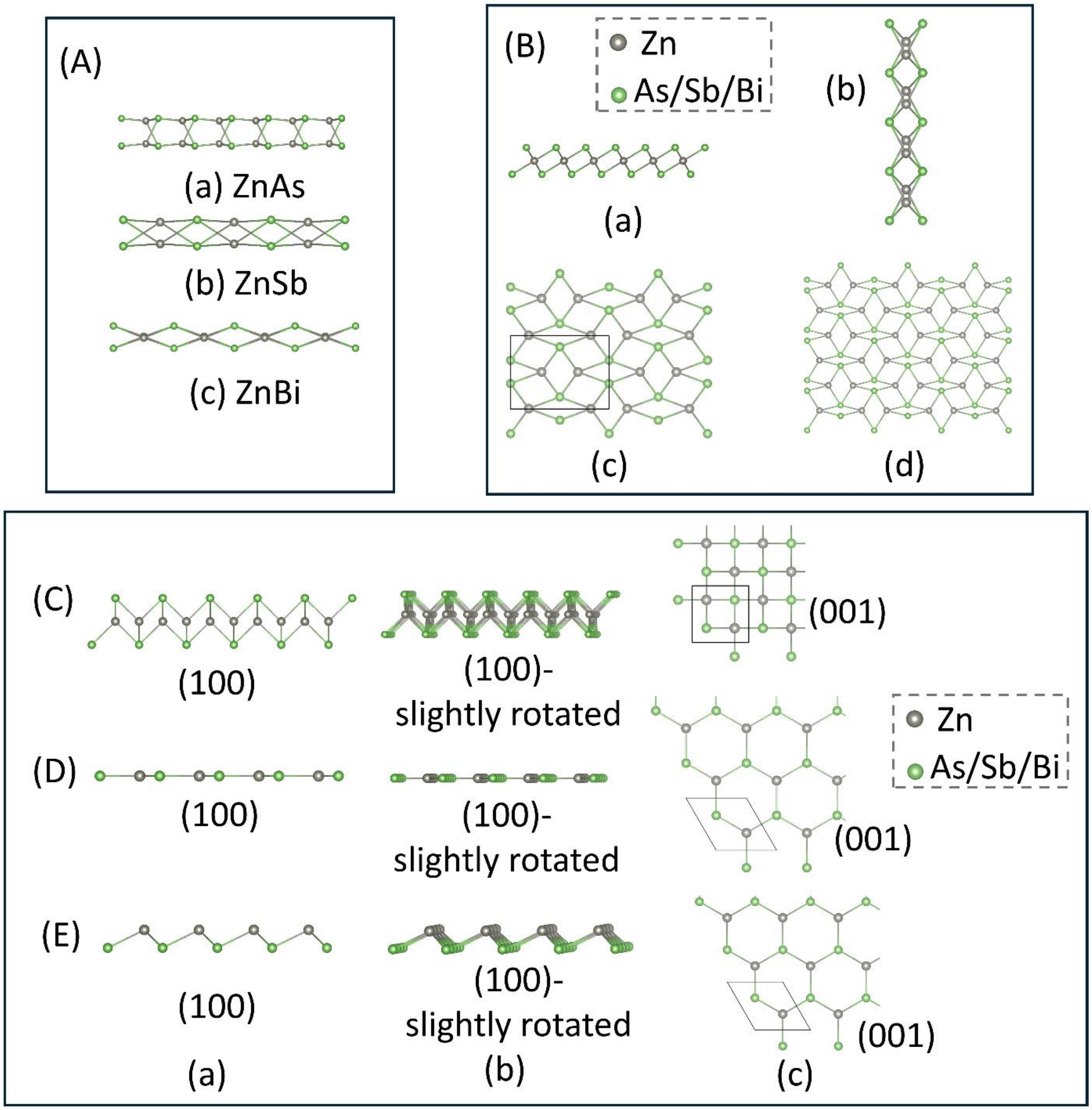

Fig. S3: Diagram representing relaxed 2D-ML structures of ZnX in (A) 2D-L2 monolayer for (a) ZnAs, (b) ZnSb, and (c) ZnBi. The geometrical distortion associated with Zn planes has been observed while going from ZnAs to ZnBi. The two Zn planes in ZnAs tend to merge into a single Zn-plane, while going from As to Bi. (B) 2D-L3 monolayer while viewing through (a) (010), (b) (100), (c) (001) and (d) (001) slightly rotated along *ac* plane invariant to *b*-axis. (C) 2D tetragonal structure with square lattice, (D) 2D hexagonal (planar honeycomb), and (E) 2D wurtzite

(puckered honeycomb). *Here, the wurtzite (puckered honeycomb) structure does not exist in 2D - ZnAs. It takes a form of planar honeycomb after full geometrical relaxation.*

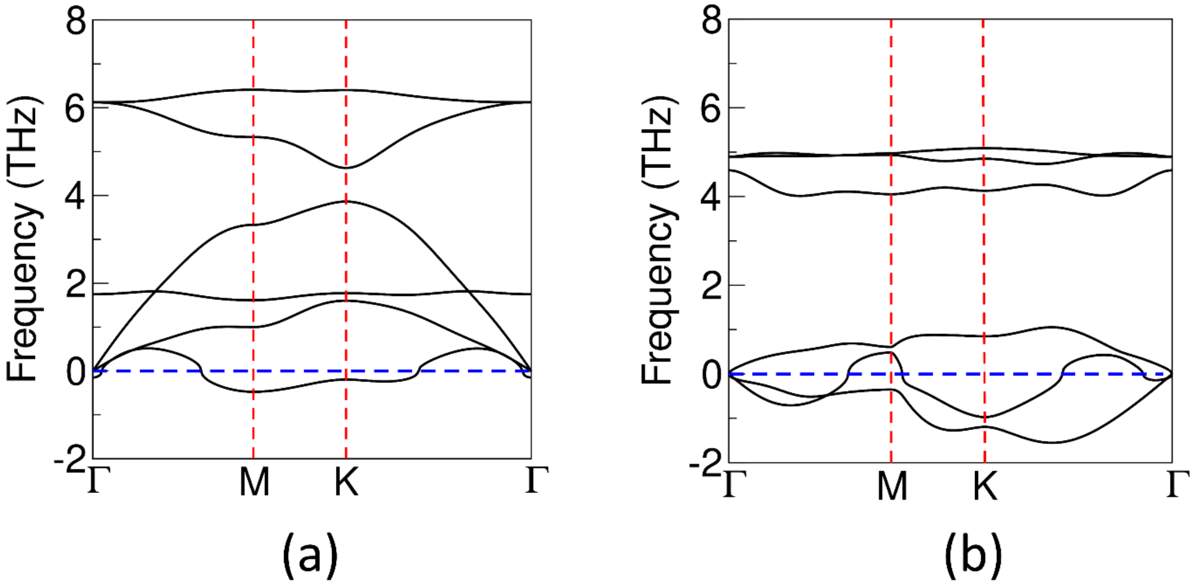

Fig. S4: Phonon dispersion curves (PHDCs) in 2D monolayers with (a) hexagonal (planar honeycomb) and (b) wurtzite (puckered honeycomb) structures in ZnSb.
